# Berberine inhibits intestinal carcinogenesis by suppressing intestinal pro-inflammatory genes and oncogenic factors through modulating gut microbiota

**DOI:** 10.1186/s12885-022-09635-9

**Published:** 2022-05-20

**Authors:** Haitao Chen, Chenxiao Ye, Biyu Cai, Fan Zhang, Xuanying Wang, Jin Zhang, Zewei Zhang, Yong Guo, Qinghua Yao

**Affiliations:** 1 Department of Integrated Chinese and Western Medicine, Institute of Basic Medicine and Cancer (IBMC), The Cancer Hospital of the University of Chinese Academy of Sciences (Zhejiang Cancer Hospital), Chinese Academy of Sciences, 310022 Hangzhou, Zhejiang China; 2 Zhejiang Chinese Medical University, 310053 Hangzhou, Zhejiang China; 3 Department of Abdominal Surgical Oncology, The Cancer Hospital of the University of Chinese Academy of Sciences (Zhejiang Cancer Hospital), 310022 Hangzhou, Zhejiang China; 4 Department of Oncology, The First Affiliated Hospital of Zhejiang Chinese Medical University, 310003 Hangzhou, Zhejiang China; 5 Key Laboratory of Traditional Chinese Medicine Oncology, Zhejiang Cancer Hospital, 310022 Hangzhou, China; 6 Key Laboratory of Head & Neck Cancer Translational Research of Zhejiang Province, 310022 Hangzhou, Zhejiang China

**Keywords:** Berberine, Colorectal cancer, Gut microbiota, Fecal microbiota transplantation, Molecular mechanism, Cytokines, NF-κB signaling pathway

## Abstract

**Background:**

The role of Berberine (BBR) in colorectal cancer (CRC) and gut microbiota has begun to appreciate. However, there was no direct evidence confirm that the gut microbiota regulated by BBR could inhibit CRC. This report investigated the effect of stool from BBR treated subjects and its effect on CRC.

**Methods:**

A mouse model for CRC was developed using azoxymethane (AOM) and dextran sulfate sodium (DSS). Intestinal tissue from affected mice were used to determine the efficacy of BBR against CRC. Stool samples were collected for the 16s rRNA gene sequencing and fecal microbiota transplantation (FMT). Finally, the mechanism of gut microbiota from BBR treated mice on CRC was explored using immunohistochemistry, RNA-Sequencing, quantitative RT-PCR, and western blot analyses.

**Results:**

BBR significantly reduced intestinal tumor development. The richness of gut microbiota were notably decreased by BBR. Specifically, the relative abundance of beneficial bacteria (*Roseburia*, *Eubacterium*, *Ruminococcaceae*, and *Firmicutes_unclassified*) was increased while the level of bacteria (*Odoribacter*, *Muribaculum*, *Mucispirillum*, and *Parasutterella)* was decreased by BBR treatment. FMT experiment determined that the mice fed with stool from BBR treated AOM/DSS mice demonstrated a relatively lower abundance of macroscopic polyps and a significantly lower expression of β-catenin, and PCNA in intestinal tissue than mice fed with stool from AOM/DSS mice. Mechanistically, intestinal tissue obtained from mice fed with stool from BBR treated AOM/DSS mice demonstrated a decreased expression of inflammatory cytokines including interleukin 1β (IL-1β), tumor necrosis factor-α (TNF-α), C-C motif chemokine 1 (Ccl1), Ccl6, and C-X-C motif ligand (Cxcl9). In addition, the NF-κB expression was greatly suppressed in mice fed with stool from BBR treated AOM/DSS mice. Real-time PCR arrays revealed a down-regulation of genes involved in cell proliferation, angiogenesis, invasiveness, and metastasis in mice fed with stool from BBR treated AOM/DSS mice.

**Conclusions:**

Stool obtained from BBR treated AOM/DSS mice was able to increase colon length while simultaneously decreasing the density of macroscopic polyps, cell proliferation, inflammatory modulators and the expression of NF-κB. Therefore, it was concluded that suppression of pro-inflammatory genes and carcinogens factors by modulating gut microbiota was an important pathway for BBR to inhibit tumor growth in conventional mice.

**Supplementary information:**

The online version contains supplementary material available at 10.1186/s12885-022-09635-9.

## Introduction

Colorectal cancer (CRC) is a major driver of cancer mortality in the world [1] with a rapidly increasing incidence and mortality in China [2]. Studies have shown that the occurrence and development of CRC were related to both genetic and epigenetic factors [3]. Presently, a growing body of evidence has suggested that the exacerbation of CRC is the effect of external environmental factors, such as smoking, dietary and other lifestyle factors contributing; and internal environment factors, characterized by immune dysfunction and the activation of tumor-related pathways [4].

Currently, studies have confirmed that the composition of the gut microbiome plays a critical role in the development of CRC. Early studies have suggested that fecal samples from patients with CRC were able to induce intestinal cancers in mice [5]. Further studies have reported some bacteria, such as *Fusobacterium nucleatum*, *Escherichia coli*, and *Bacteroides fragilis*, can promote the development of CRC [6, 7]. Conversely, the bacteria, including *Eubacterium rectale*, and *Faecalibacterium prausnitzii*, may slow the development of CRC by suppressing intestinal inflammation [8]. Therefore, modulating gut microbiota could be a viable method to treat CRC.

As an isoquinoline alkaloid, Berberine (BBR) is a compound that is extracted from *Coptis Chinensis*, *Cortex Phellode*, and *Berberis*. BBR has been suggested to treat intestinal infections in clinic [9]. At present, studies have proven that BBR can suppress colon tumorigenesis by inhibiting AMP-activated protein kinase signaling pathways and epidermal growth factor receptor (EGFR) signaling in mice [10, 11]. However, we cannot entirely explain its clinical efficacy, because its bioavailability is very low and it was poorly absorbed into the bloodstream from the intestines [12]. It is worth noting that BBR has been suggested to improve the bacterial composition in the intestine for the treatment of ulcerative colitis (UC), obesity, and atherosclerosis in mice [13–15]. These results may be associated with suppressing the inflammatory response and regulating immunity in the intestines [16, 17]. Further studies demonstrated that the inhibitory effect of gut microbiota in CRC was closely related to its metabolites. For instance, short-chain fatty acids (SCFAs) have been suggested to suppress tumors in the intestines [18, 19]. Therefore, it was suggested that BBR could potentially edit the composition of the gut microbiota to inhibit the growth of CRC.

In previous study, we had confirmed that BBR has been proved to have a moderation effect on the gut microbiota and fecal metabolites in AOM/DSS mice [20]. The current study was designed to re-examine the alteration of the gut microbiota in AOM/DSS mice when faced with BBR treatment. Additionally, we sought to investigate the effects and molecular basis of the gut microbiota altered by BBR on the progression of CRC. Through the use of carcinogen-induced conventional mouse models and FMT experiment, it was proved that suppression of pro-inflammatory genes and carcinogens factors by modulating gut microbiota was an important pathway for BBR to inhibit tumor growth in conventional mice.

## Methods

### Animals and experimental design

**Fig. 1:**
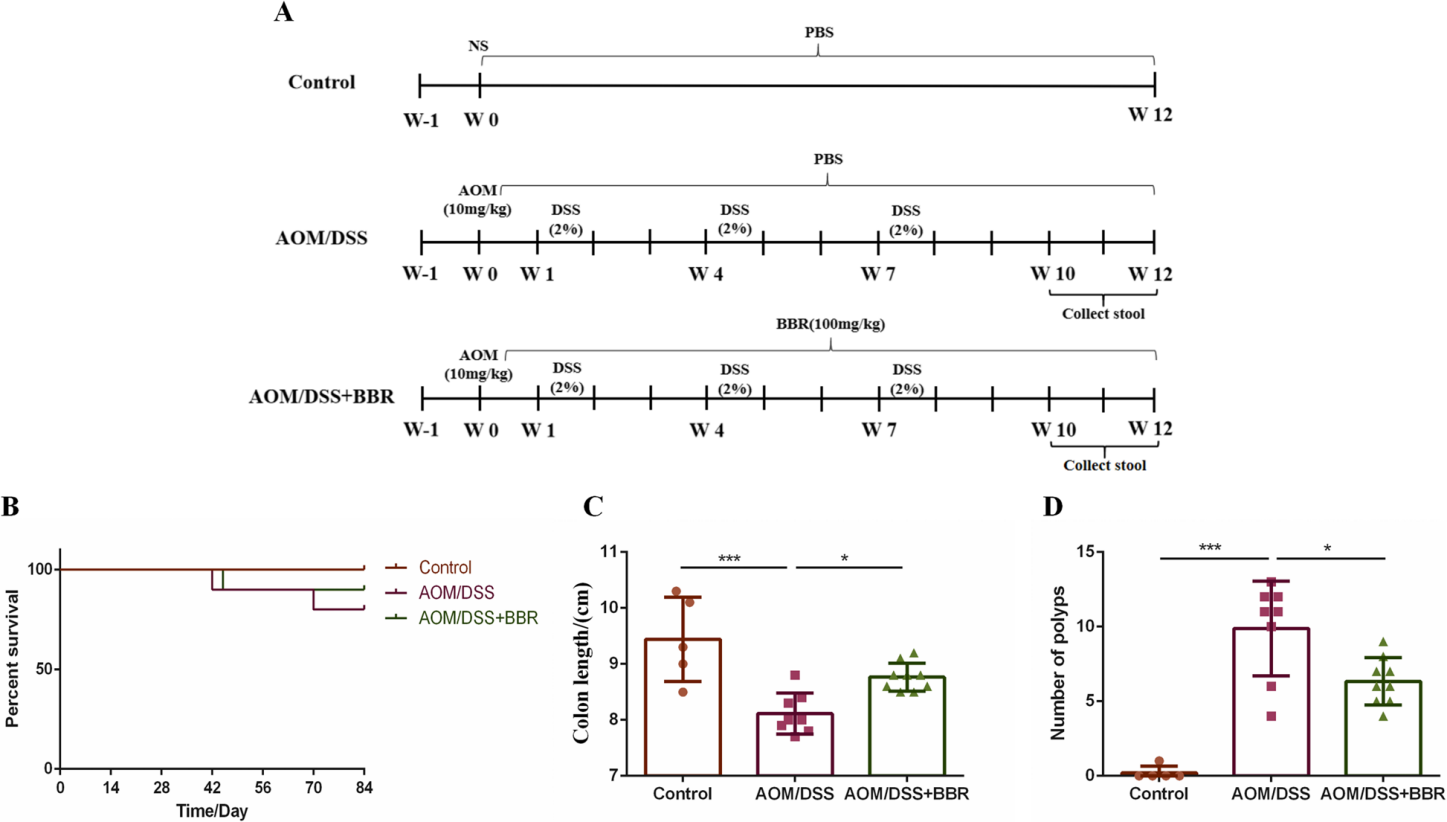
BBR inhibited intestinal tumorigenesis in an AOM/DSS mouse model. **A** Design of BBR experiment to AOM/DSS mice. **B** Survival rate of each group. **C** The colon length of each group. **D** Number of colonic polyps in each group. Data are expressed as mean ± SD (*P < 0.05, ***P < 0.001)

Female C57BL/6 mice with an average weight between 18 and 20 g were obtained from Shanghai Jihui Experimental Animal Breeding Co., Ltd. (Shanghai, China) and bred in the Laboratory Animal Center of Zhejiang Chinese Medical University (SYXK [Zhe] 2018-0012, Hangzhou, China). The procedure of AOM/DSS induced CRC model operation according to the previous study protocol [20]. After one week of adaptive based feeding, subjects were divided randomly into three groups: the control group (5 mice), the AOM/DSS group (10 mice), and the AOM/DSS + BBR group (10 mice). The AOM/DSS group and AOM/DSS + BBR group were treated with a single dose of 10 mg/kg AOM intra-peritoneally. After one week, the mice were begin given Dextran Sodium Sulfate (DSS, m.w. 36–50 kDa; MP Biomedicals) for 3 cycles (given in 2% DSS in drinking water for one week, followed by regular drinking water for two weeks). The control group received one dose of normal saline intra-peritoneally and was given unlimited access to normal drinking water. On the other hand, AOM/DSS + BBR group mice were given 100 mg/kg BBR orally daily for 12 weeks. The control group and AOM/DSS group were gavaged with normal water daily (Fig. 1A). The stool from the AOM/DSS or AOM/DSS + BBR groups were collected and stored at − 80 ℃ immediately. All mice were humanely euthanized with CO2 asphyxiation.

**Fig. 2:**
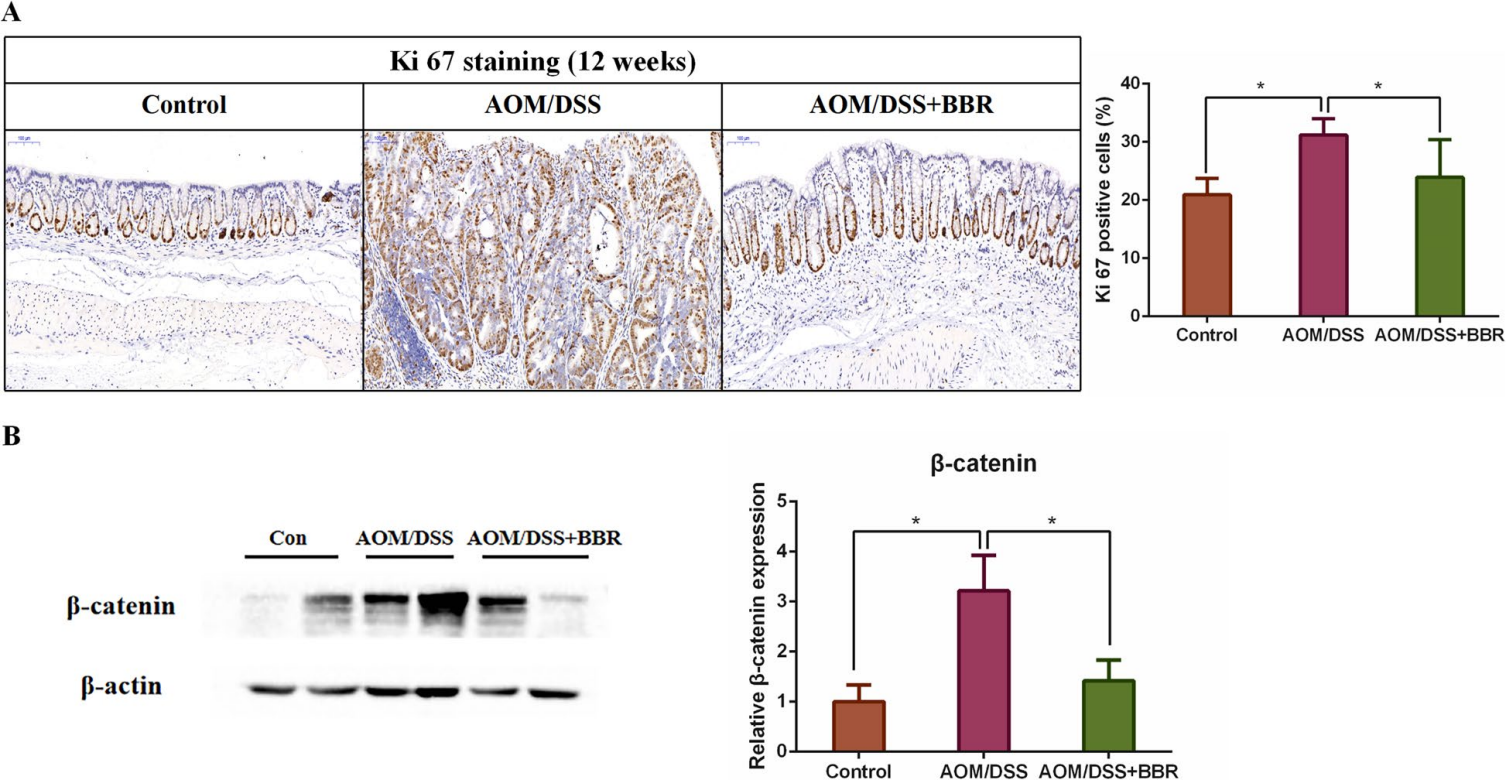
BBR suppressed colorectal carcinogenesis in AOM/DSS mouse model. **A** Immunohistochemistry staining of colorectal sections showing the expression of Ki-67 among groups. **B** Western blot analysis showing the β-catenin expression of colon tissue among groups. Data are expressed as mean ± SD (*P < 0.05)

For the FMT experiment, antibiotic pretreatment was performed as described in previous studies [5, 21]. After adaptive feeding for one week, the female C57BL/6 (18–20 g) mice were given antibiotics through drinking water containing 1 g/L metronidazole, 0.5 g/L vancomycin, 0.2 g/L ciprofloxacin, and 1 g/L neomycin daily for two weeks. After the last dose of antibiotics, the mice were randomly divided into two groups each of 10 mice: the FMT (AOM/DSS) group and the FMT (AOM/DSS + BBR) group. All mice were given one dose of 10 mg/kg AOM intra-peritoneally. After one week, the mice were begin given DSS for 3 cycles (given 2% DSS in drinking water for one week followed by normal water for two weeks). Meanwhile, all mice were gavaged daily at the first week with fecal suspension from the AOM/DSS group mice or the AOM/DSS + BBR group mice, then mice were given fecal suspension twice a week for 8 consecutive weeks (Fig. 2A).

To prepare the stool for gavage, stool samples were selected at random and mixed. Then, 350 mg of the mixed stool was suspended in 3.5 mL of PBS. The fecal slurry was centrifuged at 1000 RPM for 5 min at 4 °C, and 0.2 mL of the suspension was used for gavage into each mice. The body weight was recorded once weekly and all mice were sacrificed at 14 weeks.

### Sample collection

When the study was concluded, stool samples were collected and separately stored at -80 °C immediately for 16 S rRNA analyses. Mice were anesthetized with pentobarbital sodium. The colon was excised and washed in PBS. The colonic length and number of polyps were recorded before the colon was separated into three equivalent portions. One section of tissue was fixed in 4% formaldehyde for histological evaluation and immunohistochemistry staining. The remaining two samples were snap-frozen in liquid nitrogen and used for RNA-sequencing, PCR and western blot analysis.

### Immunohistochemistry staining

The segments were fixed in 4% formaldehyde for 24–72 h to fix the tissues and embed them in paraffin wax. After this step, tissues were cut into 4 μm thickness and mounted on glass slides. The paraffin was then removed by using citrate buffer solution (pH 6.0). Slides were stored in 3% H2O2 for 25 min and moved to normal goat serum for 1 h. Slides were then labeled with the anti-Ki 67 antibody (1:500, Abcam, USA) at 4℃ overnight, and stained with HRP-conjugated secondary antibody for 30 min. Finally, the slides were covered with DAB for several minutes, counterstained with hematoxylin, and observed using a Nikon biomicroscope device (Eclipse Ci-L). All quantification was computed using image pro plus 6.0 (Media Cybemetics, USA).

### Fecal bacterial composition analysis

The composition of microbiota in AOM/DSS with or without BBR administration was analyzed using 16 S rRNA genes analysis. The operation process of 16 S rRNA genes analysis as described by other methods as reported previously [22]. In short, DNAs were extracted from fecal samples via E.Z.N.A Stool DNA Kit (D4015, Omega, Inc., USA) according to the manufacturer’s instructions. Then, the V3-V4 region of 16 S rRNA genes was amplified with the forward primers 341 F (5’-CCTACGGGNGGCWGCAG-3’) and the reverse primer 805R (5’-GACTACHVGGGTATCTAATCC-3’). The PCR products were purified and quantified by AMPure XT beads (Beckman Coulter Genomics, Danvers, MA, USA) and Qubit ( Invitrogen, USA), respectively. Next, the Agilent 2100 Bioanalyzer (Agilent, USA) was used to prepare the amplicon pools for sequencing. The size and quantity of the amplicon library were assessed. Data was then processed using the NovaSeq PE250 platform by LC-Bio wherein FLASH was employed for the paired-end reads. The bioinformatics analysis was carried out using fqtrim(v0.94), Vsearch software(v2.3.4), DADA2, QIIME2, and R (v3.5.2) according to a previous report [22].

### PCR sequencing detection

RNA of colonic mucosae was extracted using Trizol reagent (Invitrogen, USA) following the manufacturer’s procedure [23]. The quality and purity of RNA collected were examined using a bioanalyzer 2010 and RNA 1000 Nano LabChip Kit (Agilent, USA). Finally, the cleaved RNA was reverse transcribed to generate cDNA in accordance to the protocol for RNA-Seq sample preparation kit (Illumina, San Diego, USA). Paired-end sequencing was performed on an IlluminaHiseq4000 (LC Sciences, USA).

### Quantitative reverse transcription PCR (qRT-PCR)

Total RNA of colonic tissue was extracted by using Trizol reagent. The purity and concentration of RNA were measured by spectrophotometer, and they were reverse transcribed using a complementary DNA (cDNA) conversion kit (Thermo Fisher Scientific, USA). Real-time PCR was carried out by SYBR Green master mix (Beijing ComWin Biotech Co., Ltd., China) in the 7900HT Fast Real-Time PCR system. GAPDH was used as the standard control. Primer sequences are listed in Table 1.

**Table 1:** The primers used in qRT-PCR

Primers	Forward	Reverse
IL-1b	TGCCACCTTTTGACAGTGATC	AAGGTCCACGGGAAAGACAC
TNF-α	TGACTCCAAAGTAGACCTGC	CTACTCCCAGGTTCTCTTCA
Cxcl9	AACGGAGATCAAACCTGCCT	AGATTCAGGGTGCTTGTTGGT
Mmp9	CGACTTTTGTGGTCTTCCCC	CTTCTCTCCCATCATCTGGGC
Muc16	GCTCAGCACATCGACACAGA	CTCGTGCCTTTCATAGCAGC
Ereg	ACATGGACGGCTACTGCTTG	AAGTGCTCACATCGCAGACC
Gapdh	GCAGAGTGTTTCCTCGTCCC	ACTGTGCCGTTGAATTTGCC

### Western blot

Tissues samples of colonic segments were lysed in RIPA lysis buffer (Upstate, USA) and the concentration of proteins was determined with a Pierce™ BCA Protein Assay Kit (Beyotime BiotechnologyCo., Ltd., China). The protocol of Western blot was carried out as described previously [4]. Briefly, proteins were incubated in 10% SDS-PAGE and transferred to polyvinylidene fluoride (PVDF) membranes. The membranes were incubated overnight in a primary antibody solution diluted as follows: β-catenin (1:1000), PCNA (1:1000), IL-1b (1:1000), TNF-α (1:1000), NF-κB (1:1000), and β-actin (1:3000). Then, incubation with HRP-conjugated antibodies was conducted for 2 h. The antibody-antigen complexes were measured via the ECL Plus Western Blot Detection Reagents (Biosharp, China). Due to the blots cut prior to hybridisation with antibodies, the images showing full-length membranes with edges visible were cannot be provided. So the images of all blots can be viewed in the Supplementary information.

### Statistical analysis

All values were expressed as mean ± S.D. The heatmap was made via the online website (https://www.omicstudio.cn/login). Differences between the two groups were analyzed by unpaired Student’s t-test. The differences between multiple groups were analyzed by one-way ANOVA using GraphPad Prism 6.01. *P* value of < 0.05 was considered statistically significant.

## Results

### BBR inhibited intestinal tumorigenesis and colonocyte proliferation in an AOM/DSS mouse model

To determine the role of BBR on intestinal tumorigenesis and colonocyte proliferation, we gavaged BBR to AOM/DSS induced CRC mice (Fig. 1A). The survival curve suggested that the mortality of subjects receiving AOM/DSS was greater than that of all others (Fig. 1B). As expected, AOM/DSS had a significant effect on colon length. In comparison to the control, the colonic length was shorter in the AOM/DSS group (*p* < 0.05, Fig. 1C). However, the colon length was significantly increased after BBR administration (*p* < 0.05, Fig. 1C). Additionally, AOM/DSS mice showed significantly greater numbers of colonic polyps than the control group (*p* < 0.001, Fig. 1D). On the contrary, after BBR treatment, the number of polyps was remarkably reduced (*p* < 0.05, Fig. 1D).

Furthermore, immunohistochemistry staining and western blot analysis respectively confirmed that AMO/DSS significantly increased the expression of Ki-67 and β-catenin when compared to the control. However, the expression was remarkably reduced after BBR treatment (Fig. 2A-B). Therefore, these data indicated that BBR inhibited colonic epithelial cell proliferation in AOM/DSS mice.

### BBR modulated the gut microbiota in AOM/DSS mice

**Fig. 3:**
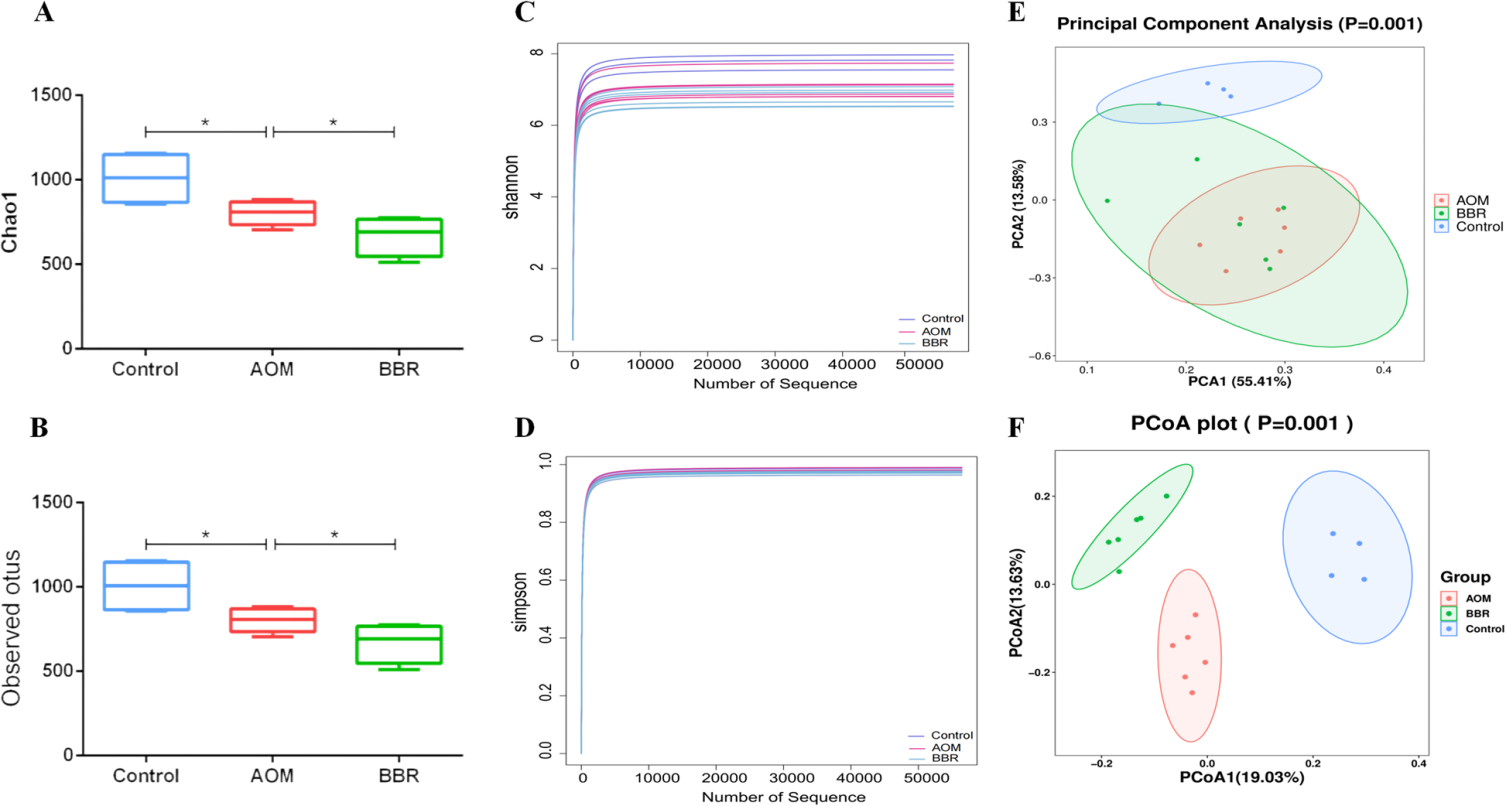
Diversity and composition of gut microbiota among groups. **A**, **B** The index of Chao1 and observed otus of gut microbiota. **C**, **D** The index of Shannon and Simpson of gut microbiota. **E**, **F** Unsupervised PCA scatter plot and weighted uniFracbased PCoA of feces microbiota

To examine the role of BBR on the diversity and structure of the gut microbiota, 16 S rRNA gene sequencing was performed on stool collected from experimental mice. Compared to the AOM/DSS group, the indexes of Chao 1 and Observed otus were reduced significantly after BBR (*p* < 0.05, Fig. 3A-B), which was consistent with a previous study [20]. In addition, no statistical difference was found between the Shannon and Simpson indexes of the control and AOM/DSS group. The BBR also showed no effect on the Shannon and Simpson indexes in AOM/DSS induced CRC mice, indicating that the diversities of gut microbiota were not impacted by AOM/DSS and BBR intervention (*p* > 0.05, Fig. 3C-D). However, the principal component analysis (PCA) and UniFrac principal coordinate analysis (PCoA) demonstrated that AOM/DSS induced CRC mice had significantly distinct gut microbiota composition compared to the control group, and BBR administration restored the disturbance of microbial composition structure in AOM/DSS mice (Fig. 3E-F).

**Fig. 4:**
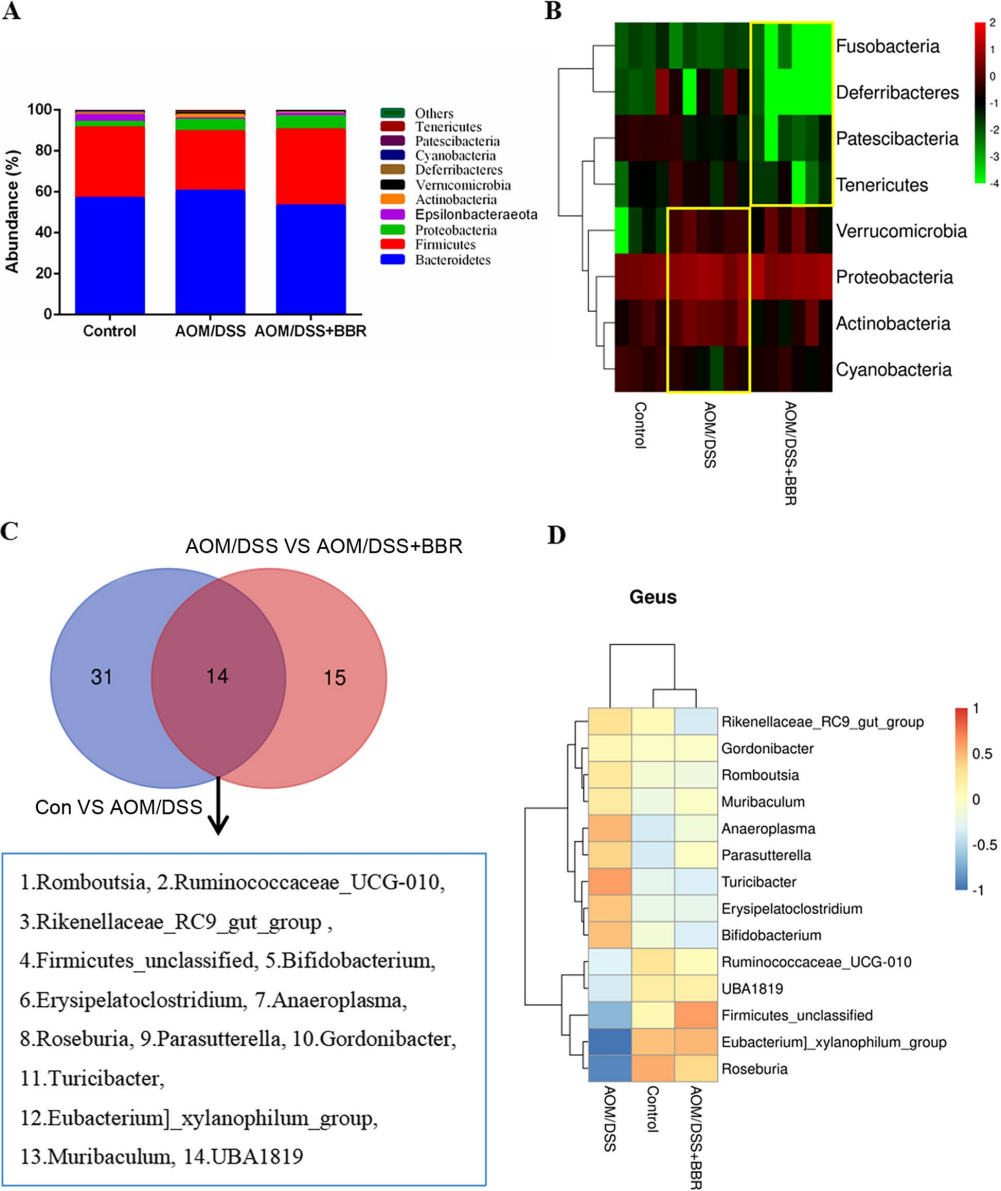
BBR regulated the imbalance of gut microbiota in AOM/DSS mice. **A** The top 10 relative abundances of gut microbiota at phylum. **B** Heat map of relative abundances of bacterial phyla level among groups. **C** Venn diagrams demonstrate the number of altered bacterial genera shared between control and AOM/DSS (blue), AOM/DSS and AOM/DSS+BBR group (red). **D** Heat map of relative abundances of bacterial genera level among groups

The top 10 relative abundances of the gut microbiota were presented (Fig. 4A). Previous reports indicated that an imbalance of the gut microbiome was found in AOM/DSS induced CRC mice [24]. In our study, we also found the disorders of the gut microbiota in AOM/DSS mice. Specifically, the concentration of *Actinobacteria*, *Verrucomicrobia*, and *Proteobacteria* increased and *Cyanobacteria* decreased after AOM/DSS intervention in the phylum level. Furthermore, the abundance of *Actinobacteria* was restored following BBR treatment, and the concentration of *Fusobacteria*, *Patescibacteria*, *Deferribacteres*, and *Tenericutes* decreased remarkably (Fig. 4B). At the genus level, we found that 14 bacterial genera significantly changed after AOM/DSS and BBR intervention (Fig. 4C). Specifically, compared with the control group, AOM/DSS induced CRC mice showed a significant increase in the relative abundances of *Muribaculum*, *Turicibacter*, *Romboutsia*, *Rikenellaceae_RC9_gut_group*, *Bifidobacterium*, *Anaeroplasma*, *Parasutterella*, *Erysipelatoclostridium*, *Gordonibacter*, and a decrease in *Roseburia*, *Ruminococcaceae_UCG-010*, *Firmicutes_unclassified*, *UBA1819*, and *Eubacterium]_xylanophilum_group*. Most of these species were remarkably restored after BBR administration. Furthermore, BBR intervention also could inhibit the level of pathogenic bacteria (such as *Odoribacter*, *Mucispirillum*, *Peptococcus*, and *Candidatus_Saccharimonas*) while stimulating the relative abundance of some SCFAs producing bacteria (such as *Ruminococcaceae_UCG-004*, Alistipes, *Ruminococcaceae_unclassified*, and *Ruminococcaceae_UCG-005*) (Fig. 4D).

**Fig. 5:**
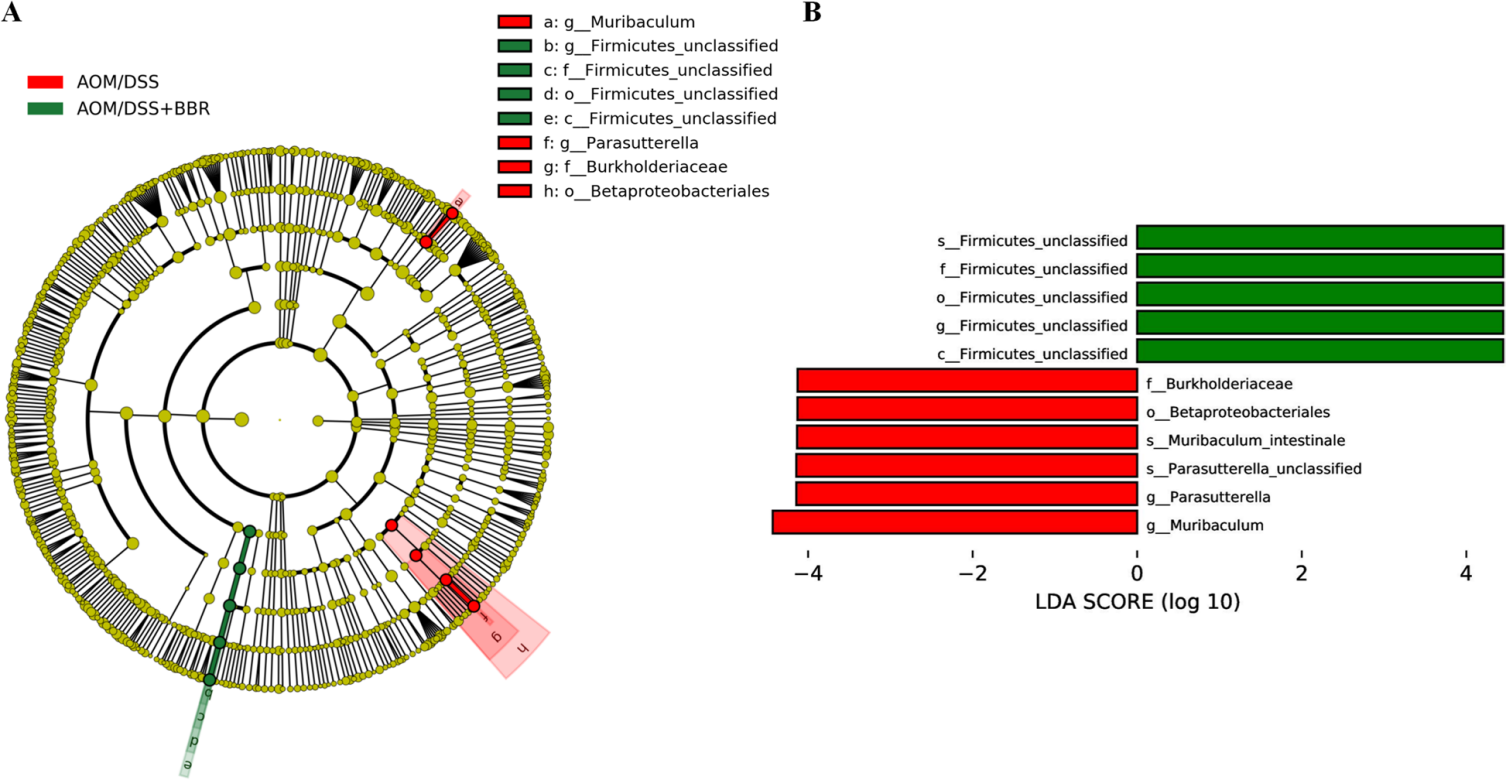
BBR modulated gut microbiota community in AOM mice. **A** LEfSe analysis biomarker taxa. **B** LEfSe analysis histogram

Moreover, the LEfSe method was used to find biomarkers between AOM/DSS and AOM/DSS + BBR group. Compared to the AOM/DSS group, the AOM/DSS + BBR group displayed a predominance of *s_Firmicutes_unclassified*, *f_Firmicutes_unclassified*, *o_Firmicutes_unclassified*, *g_Firmicutes_unclassified*, and *c_Firmicutes_unclassified*, whereas the AOM/DSS group exhibited a predominance of *g_Muribaculum*, *g_ Parasutterella*, *s_Parasutterella_unclassified*, *s_Muribaculum_intestinale*, *o_Betaproteobacteriales*, and *f_Burkholderiaceae* (Fig. 5A-B). In summary, these findings suggest that BBR improved the degree of dysbiosis in AOM/DSS mice and that *Firmicutes_unclassified* and *Muribaculum* can be used as identification biomarkers between the AOM/DSS and AOM/DSS + BBR groups.

### Stool from BBR treated mice inhibited the progression of tumor in an AOM/DSS mouse model

**Fig. 6:**
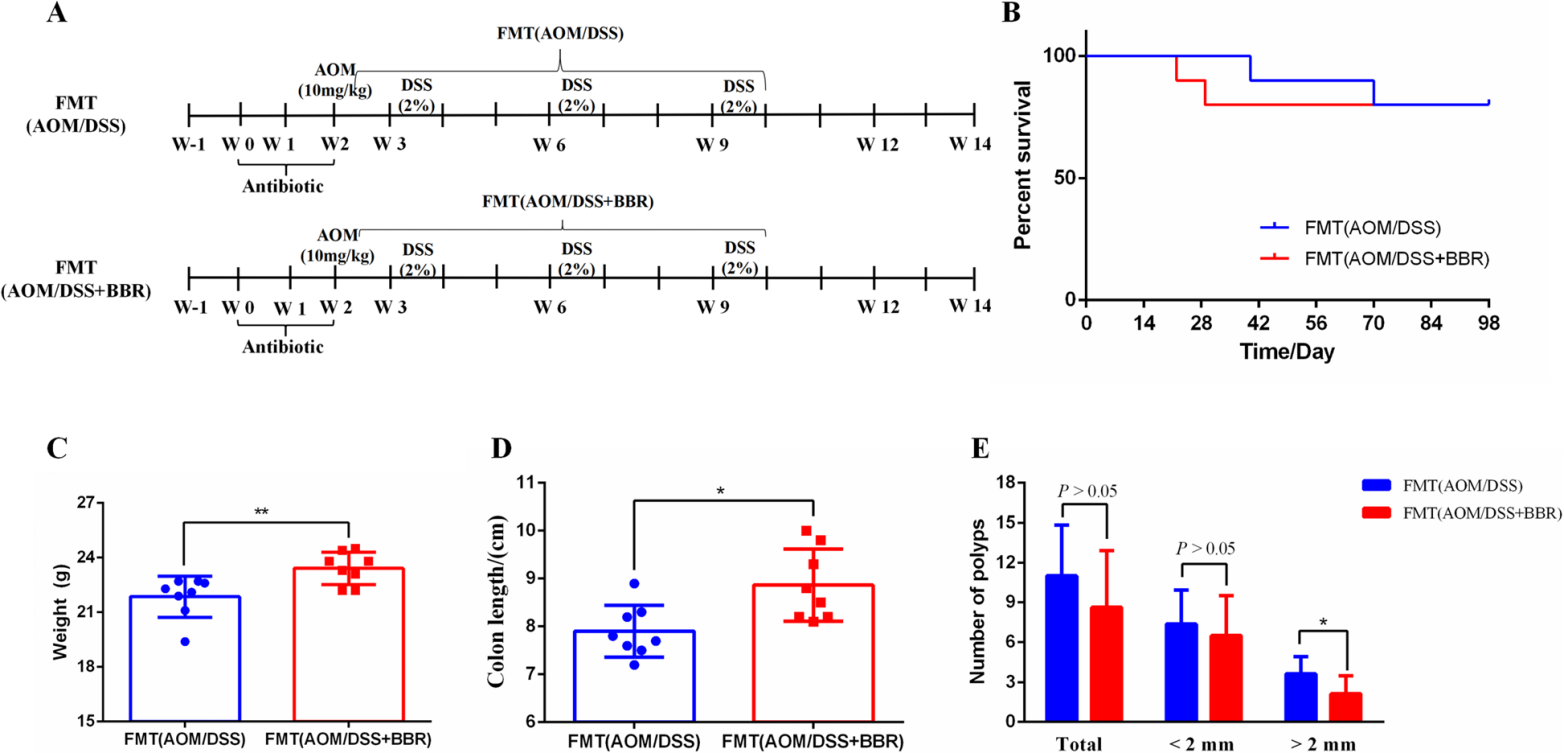
Stool from BBR treated mice inhibited intestinal tumorigenesis in an AOM/DSS mouse model. **A** Design of FMT experiment to AOM/DSS mice. **B** Survival rate of each group. **C** Body weight of mice. **D** Colon length of mice. **E** Number of colonic polyps in each group. Data are expressed as mean ± SD (*P < 0.05, **P < 0.01)

Stool gavaged from AOM/DSS or AOM/DSS + BBR mice was hypothesized to have a effect on the intestinal tumorigenesis and colonocyte proliferation, we gavaged fecal suspension from the AOM/DSS mice or AOM/DSS + BBR mice to AOM/DSS mice respectively (Fig. 6A). The survival curve showed no difference in mortality between the FMT (AOM/DSS) and FMT (AOM/DSS + BBR) mice (Fig. 6B). Nevertheless, the FMT (AOM/DSS + BBR) mice showed a higher body weight than those in the FMT (AOM/DSS) mice at the 12th week (*p* < 0.05, Fig. 6C). Meanwhile, the colonic length in FMT (AOM/DSS + BBR) mice was longer compared to the FMT (AOM/DSS) mice. Although no difference was observed in the total number of colonic polyps within the two groups, the number of colonic polyps with a diameter of > 2 mm in the FMT (AOM/DSS + BBR) group lower than that of in the FMT (AOM/DSS) group (*p* < 0.05, Fig. 6D).

**Fig. 7:**
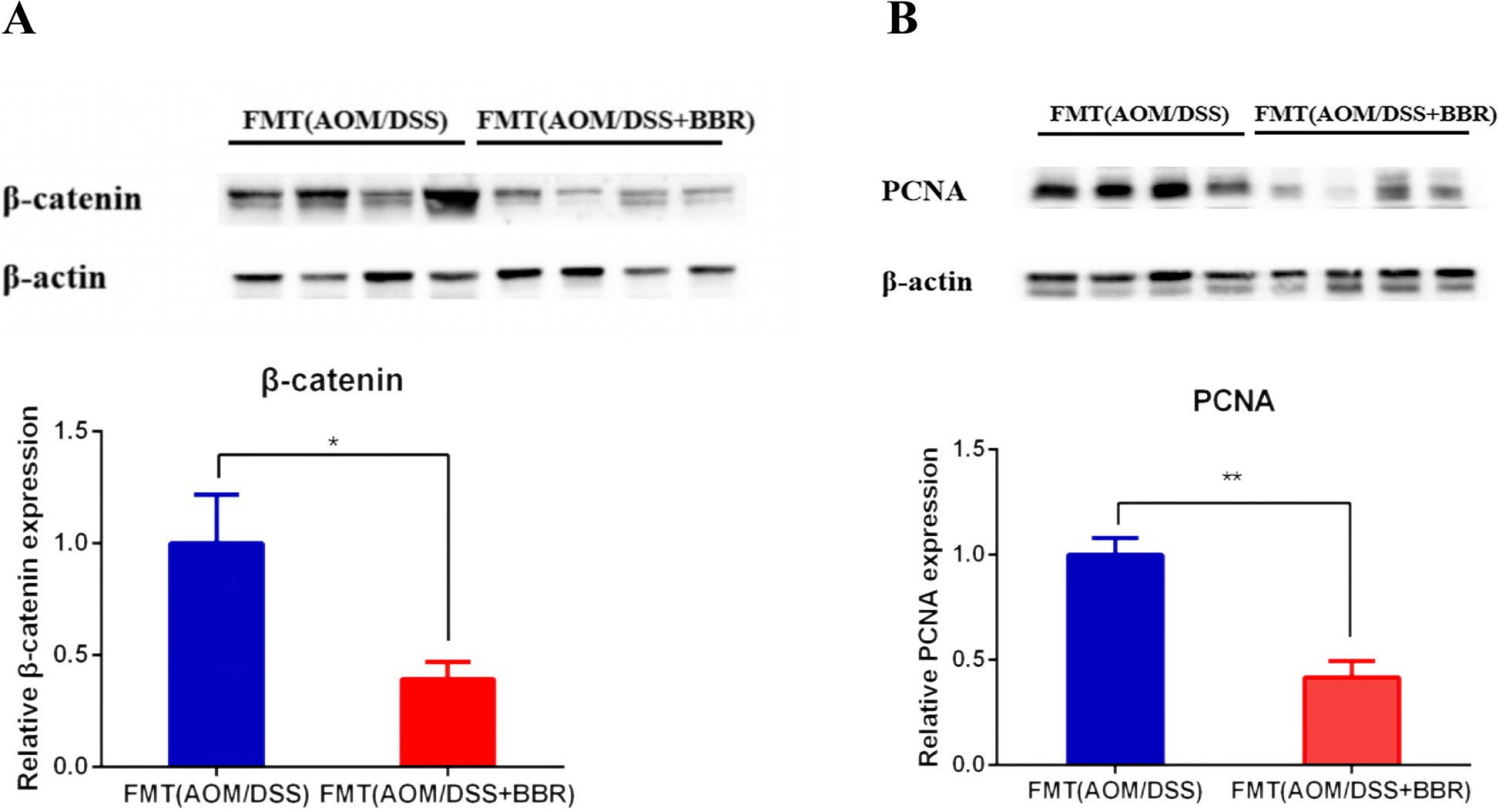
Stool from BBR treated mice suppressed colorectal carcinogenesis in an AOM/DSS mouse model. **A**-**B** Western blot analysis showing the β-catenin and PCNA expression of colon tissue among groups. Data are expressed as mean ± SD (*P < 0.05, **P < 0.01)

Moreover, western blot showed a decreased PCNA and β-catenin protein expression in the FMT (AOM/DSS + BBR) group compared to the FMT (AOM/DSS) group (*p* < 0.05, Fig. 7A-B). Taken together, this data suggested that gut microbiota from BBR treated mice inhibited the colonic tissue cell proliferation in intestinal tumorigenesis.

### Stool from BBR treated mice suppressed the pro-inflammatory factors infiltration in AOM/DSS mice

**Fig. 8:**
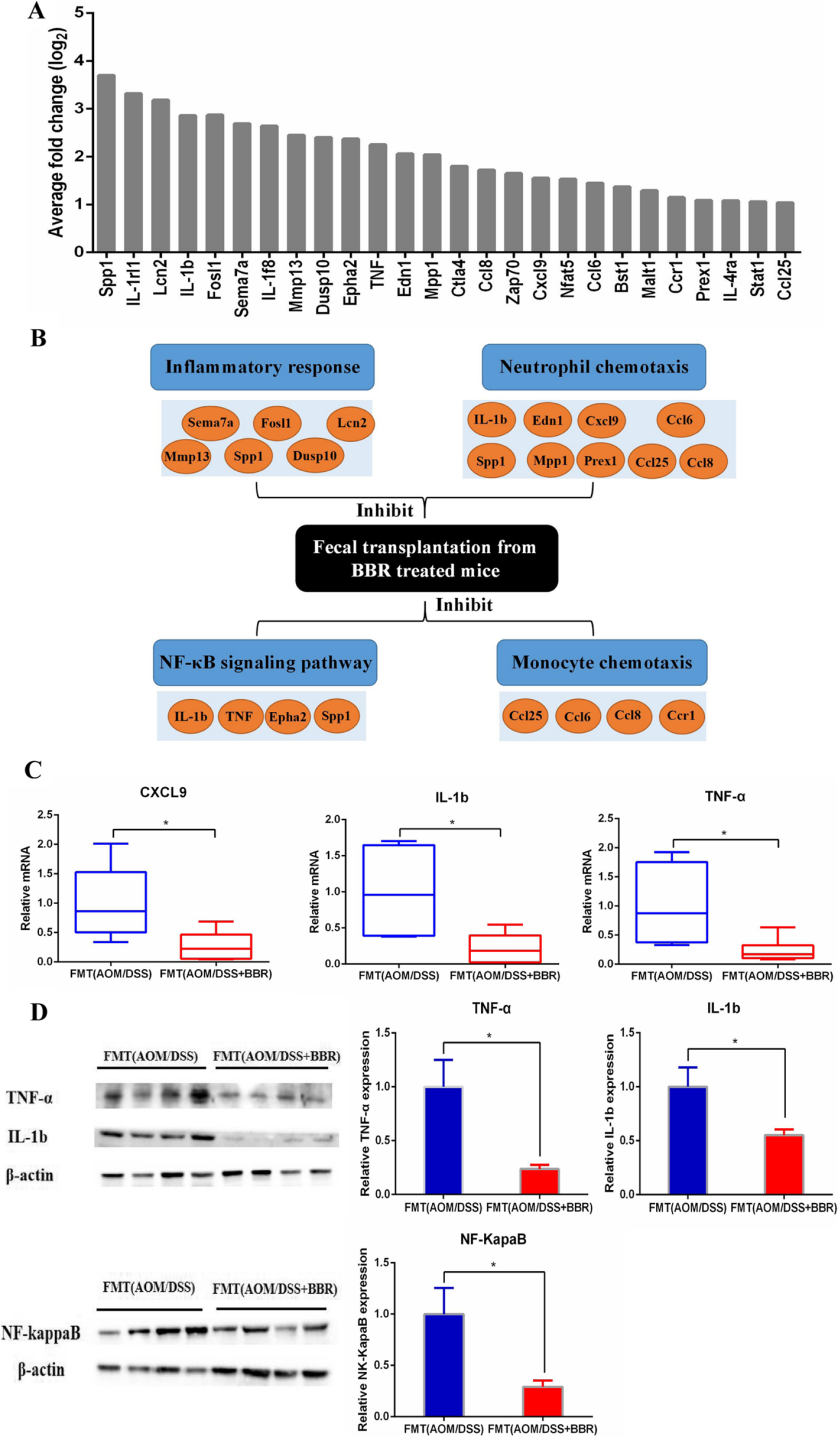
Stool from BBR treated mice decreased the expression of pro-inflammatory genes infiltration in AOM/DSS mice. **A** Significant downregulation in the expression of 26 transcripts involved in inflammatory response by RNA-Sequencing after gavage of BBR treated stool to an AOM mouse model. **B** A systematic diagram showing major inflammatory pathways implicated by down-regulated genes identified by RNA-Sequencing. **C** qRT-PCR was performed to verify the changes in expression of genes including Cxcl9, IL-1b, and TNF-α. **D** Western bolt was performed to confirm the changes in expression of genes including IL-1b, TNF-α and NF-κB (*P < 0.05)

To obtain a greater understanding into the inhibitory effect of the gut microbiota from BBR treated mice on CRC, we detected the gene expression profile of colonic cells at 14 weeks after gavage fecal suspension in CRC model mice using RNA-Sequencing. The differential genes involved in inflammatory response were identified with |logFC| >1 and adjust *P* value < 0.01. 26 genes were involved in inflammatory response and changed significantly between the two groups (Fig. 8A). Compared to the FMT(AOM/DSS) mice, the expression of genes chemokine C-C motif ligands 1 (Ccl1), 6 (Ccl6), 8(Ccl8), and C-X-C motif ligand 9 (Cxcl9) was decreased remarkably in the FMT(AOM/DSS + BBR) mice. The list also included the genes interleukin 1b (IL-1b), tumor necrosis factor (TNF), and Eph receptor A2 (EphA2), which can promote the NF-κB signaling pathway, were also decreased in FMT(AOM/DSS + BBR) mice. Meanwhile, semaphorin 7 A (Sema7a), matrix metallopeptidase 13 (Mmp13), dual-specificity phosphatase 10 (Dusp10), were reduced significantly in FMT(AOM/DSS + BBR) mice. Overall, these genes were associated with the immune system involving inflammatory response, chemotaxis, and NF-κB signaling pathway (Fig. 8B). Quantitative RT-PCR was performed on genes identified in the RNA-sequencing study by using specific primer-probes confirmed the changes in expression of IL-1b, TNF-α, and Cxcl9 (Fig. 8C). Furthermore, the protein expression of IL-1b, TNF-α, and NF-κB in the colonic tissue was also suppressed in the FMT(AOM/DSS + BBR) mice (Fig. 8D).

### Stool from BBR treated mice decreased expression of oncogenic factors in AOM/DSS mice

**Fig. 9:**
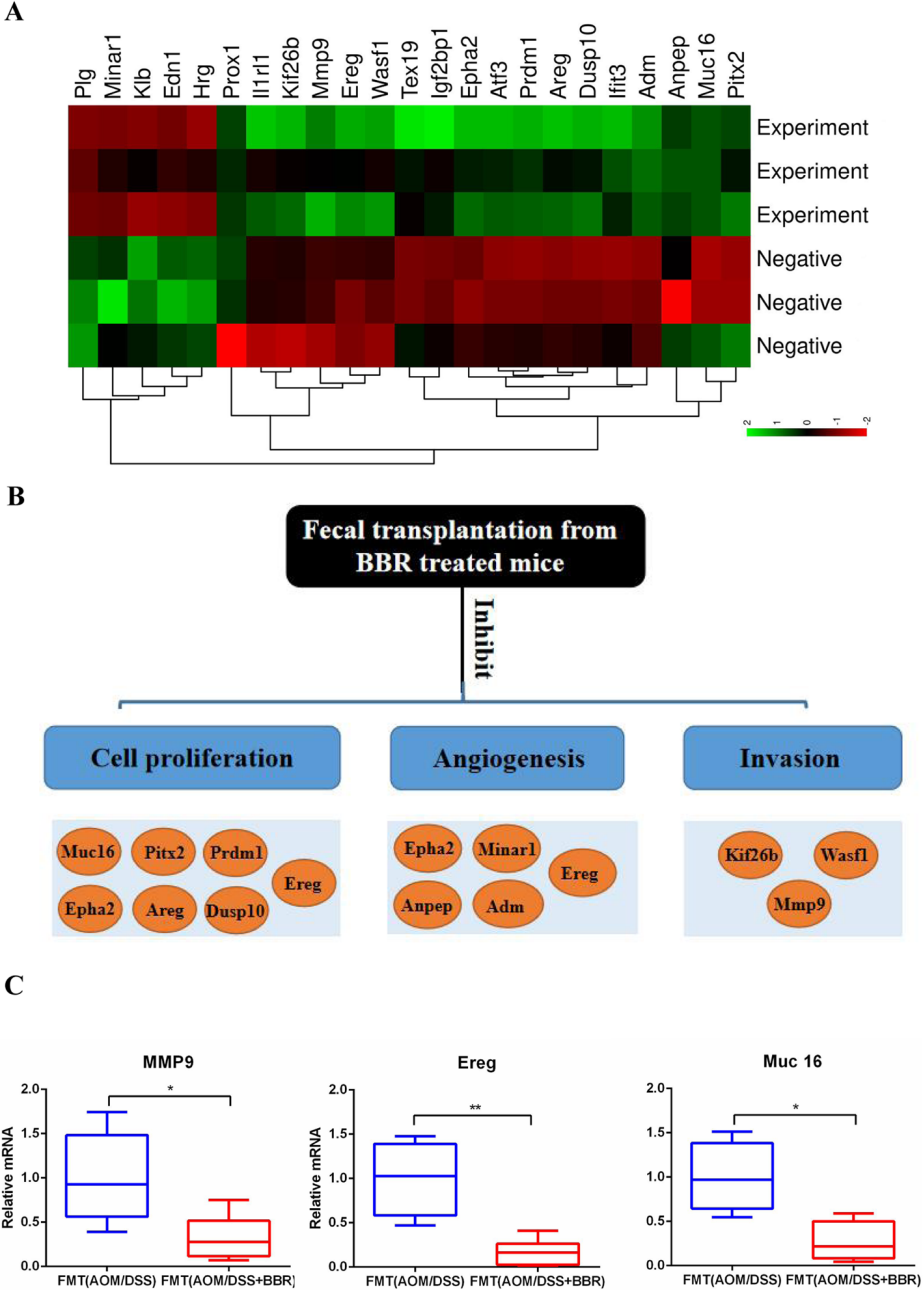
Stool from BBR treated mice decreased expressions of oncogenic factors in AOM/DSS mice. **A** Significant changes in the expression of 23 transcripts by using RNA-Sequencing. **B** A systematic diagram showing major biological pathways of tumorigenesis and down-regulated genes detected by the RNA-Sequencing. **C** qRT-PCR validation was performed to confirm changes in expression of genes including Muc16, Ereg, and MMP (*P < 0.05, **P < 0.01)

According to the RNA-Sequencing, the expression of genes involved in cancer pathways were profiled by using colonic tissue at 14 weeks after gavage fecal suspension in the CRC model. To identify the differential genes involved in tumorigenesis, the criteria of |logFC| > 2 and adjust *P* value < 0.05 were used for screening. This result found 23 genes representing major biological pathways with major differences between the two groups. Results showed that 5 genes were up-regulated and 18 genes were down-regulated in the FMT (AOM/DSS + BBR) group compared to the FMT (AOM/DSS) group (Fig. 9A). These genes were related to oncogenic pathways, including cell proliferation, angiogenesis, and invasion (Fig. 9B). Several down-regulated genes including matrix metallopeptidase 9 (Mmp9), epiregulin (Ereg), and mucin 16 (Muc16) were confirmed by qRT-PCR using specific primer-probes (Fig. 9C).

## Discussion

Modern studies have suggested that the there is a symbiotic relationship between humans and gut gut microbiota. Several reports have also suggested the role of the gut microbiota in the progression of CRC. Specifically, it was suggested that an increase in *Actinobacteria* [25], *Fusobacteria* [26], and *Tenericutes* [27] were relevant to the progression of CRC, demonstrating that modulating the composition of gut microbiota have potential therapeutic effects on CRC. Fortunately, a growing body of evidence has suggested that BBR is able to suppress the growth of CRC through regulation of the gut microbiota. However, the overall conclusion is still unclear. As such, this study functions to provide evidence to support the inhibitory role of the BBR treated microbiome on CRC in conventional mouse models.

Emerging research has highlighted the inhibitory role of BBR on the pathogenesis of CRC. Prior studies have hinted at the association between BBR and CRC through signaling pathways such as the NF-κB pathway, EGFR signaling, and Wnt signaling pathways [10, 11, 28]. Consistently, by assessing the tumor growth and number of polyps in AOM/DSS mice treated with BBR, it was suggested that BBR was able to inhibit the pathogenesis of CRC. Our previous study has suggested that BBR was able to module the enteric microbiome and fecal metabolites in AOM/DSS induced CRC mice [20]. In this study, BBR was found to decrease the richness of the microbiota without affecting its diversity, which was in parallel to previous reports [20]. Further analysis has confirmed that BBR significantly decreased the relative concentration of *Actinobacteria Fusobacteria*, *Patescibacteria*, *Deferribacteres*, and *Tenericutes* significantly at the phylum level. These changes of gut microbiota were associated with inhibition of CRC. In the aforementioned study, the abundance of *Actinobacteria*, *Tenericutes*, and *Fusobacteria* were associated with CRC and were amplified during colorectal carcinogenesis [29]. Similarly, the abundance of *Tenericutes* and *Deferribacteres* was also elevated and suggested increased inflammation in the colitis model [30, 31]. Consequently, these changes of gut microbiota caused by BBR treatment provide a guideline for future studies.

The findings obtained herein indicate that BBR inhibits CRC and regulates the microbiome in AOM/DSS mice. It is currently unknown whether this interaction is direct or indirect. Although there was no difference in mortality, the lower polyp (> 2 mm) number in mice fed with stool from AOM/DSS + BBR group, confirm that role of the gut microbiota treated by BBR on CRC. Additionally, the PCNA and β-catenin protein expression decreased significantly in stool from AOM/DSS + BBR group mice, as well as the expression of proliferation-related genes including Muc16, Mmp9, and Ereg. This decrease in proliferation genes expression can be biologically related to the inhibition of tumorigenesis.

FMT is an effective method and is important to the anti-tumor mechanistic study of microbiota treated with BBR in AOM/DSS mice. Prior research has suggested that various types of bacteria have diverse effects on colorectal carcinogenesis. For instance, *Mucispirillum* and *Odoribacter*, enriched in the AOM/DSS induced CRC mice, which can promote intestinal inflammatory response and tumor progression [32, 33]. *Muribaculum*, which bloomed in T cell-induced colitis mice, was associated with the progression of the intestinal inflammatory response [34]. On the contrary, *Eubacterium* [35], *Ruminococcaceae* [36], *Alistipes* [37], and *Roseburia* [38] could produce short-chain fatty acids, which had an inhibitory effect of inflammation and anti-neoplastic properties. Compared to AOM/DSS mice, stool from AOM/DSS + BBR group had more *Eubacterium*, *Ruminococcaceae*, *Alistipes, Roseburia*, *and Firmicutes_unclassified*, along with low *Mucispirillum*, *Odoribacter*, *Turicibacter*, and *Parasutterella* that can form a symbiotic bacterial network to inhibit the progression of CRC. Although this result defers from previous studies, [20] the result may be related to the different animal batches, feeding time, and drug dosage. It also can be concluded that BBR treatment effectively inhibited the abundance of pathogenic bacteria and conditional pathogens while promoting the growth of short-chain fatty acid-producing bacteria in AOM/DSS induced CRC mice. Notably, it is critical to consider the effects of a multi-microbial complex rather than an individual bacterium in our model. Therefore, this results offers unique data on the in-vivo effects of a multi-microbial complex from BBR treated mice and shows that the microbiota treated by BBR is inhibited colorectal carcinogenesis compared to those without BBR treatment. Besides, we consider that the reason why the therapeutic effect of gut microbiota is worse than the direct anti-tumor effect of BBR may be related to the multiple pathways of BBR on CRC.

To elucidate the molecular mechanism of gut microbiota on inhibition of CRC, our transcriptome data suggests that the stool from BBR treatment mice may inhibit serveral inflammatory and oncogenic pathways in CRC. Decreased expression of IL-1b and TNF-α was observed, suggesting that the NF-κB plays a major role to inhibit CRC by the BBR treated microbiome. Many studies have shown have shown that the NF-κB pathway is involved in colorectal tumorigenesis. Meanwhile, IL-1b and TNF-α, as stimulators of NF-κB pathway, both enhanced tumorigenesis in CRC [39, 40], whereas blockade of the NF-κB pathway inhibited tumor growth [41]. Interestingly, recent advances have reported that FMT ameliorated intestinal inflammation and protected the epithelium by regulating the TLR-MyD88-NF-κB signaling pathway in mice with CRC [42]. Contrarily, *Fusobacterium nucleatum* increased the proliferation of colorectal cancer cells via activating the Toll-like receptor 4 signaling to NF-κB and miR21 [43]. Although the taxonomical resolution to the strain level was insufficient, this data also showed that the gut microbiota from BBR treated AOM/DSS mice decreased the expressions of genes in the NF-κB signaling pathway. Therefore, our results emphasize the importance of the NF-κB pathway inhibited by BBR treated microbiota.

In addition, the down-regulation of Ccl1, Ccl2, and Cxcl9 genes suggested the importance role of the microbiome in immune cell chemotaxis. Consistently, previous literature showed that gut microbiota could directly modulate tumor microenvironment in CRC [44]. Moreover, the transcriptome data showed decreased several proliferation genes expressions in the FMT (AOM/DSS + BBR) mice, such as Muc16 which can promote proliferation via JAK2/STAT3 [45] and DUSP10 which can regulate ERK1/2-KLF5 to stimulate cell proliferation [46]. Furthermore, our results also showed that the expression of genes involved the angiogenesis, invasion, and metastasis were decreased significantly in FMT (AOM/DSS + BBR). For example, Muc16 can increase migration and invasion through interaction with mTOR [45]. EphA2 is a poor prognostic marker in stage II/III CRC and can promote cell migration and invasion in colorectal cancer [47]. Besides, the study also found that up-regulation of Ereg expression by promoter demethylation might be an important way in activating the EGFR pathway in the pathogenesis of CRC [48]. Hence, the results indicated that the microbiome from BBR treated AOM/DSS mice have inhibitory effects on CRC by regulating cell proliferation, angiogenesis, and invasion/metastasis.

**Fig. 10:**
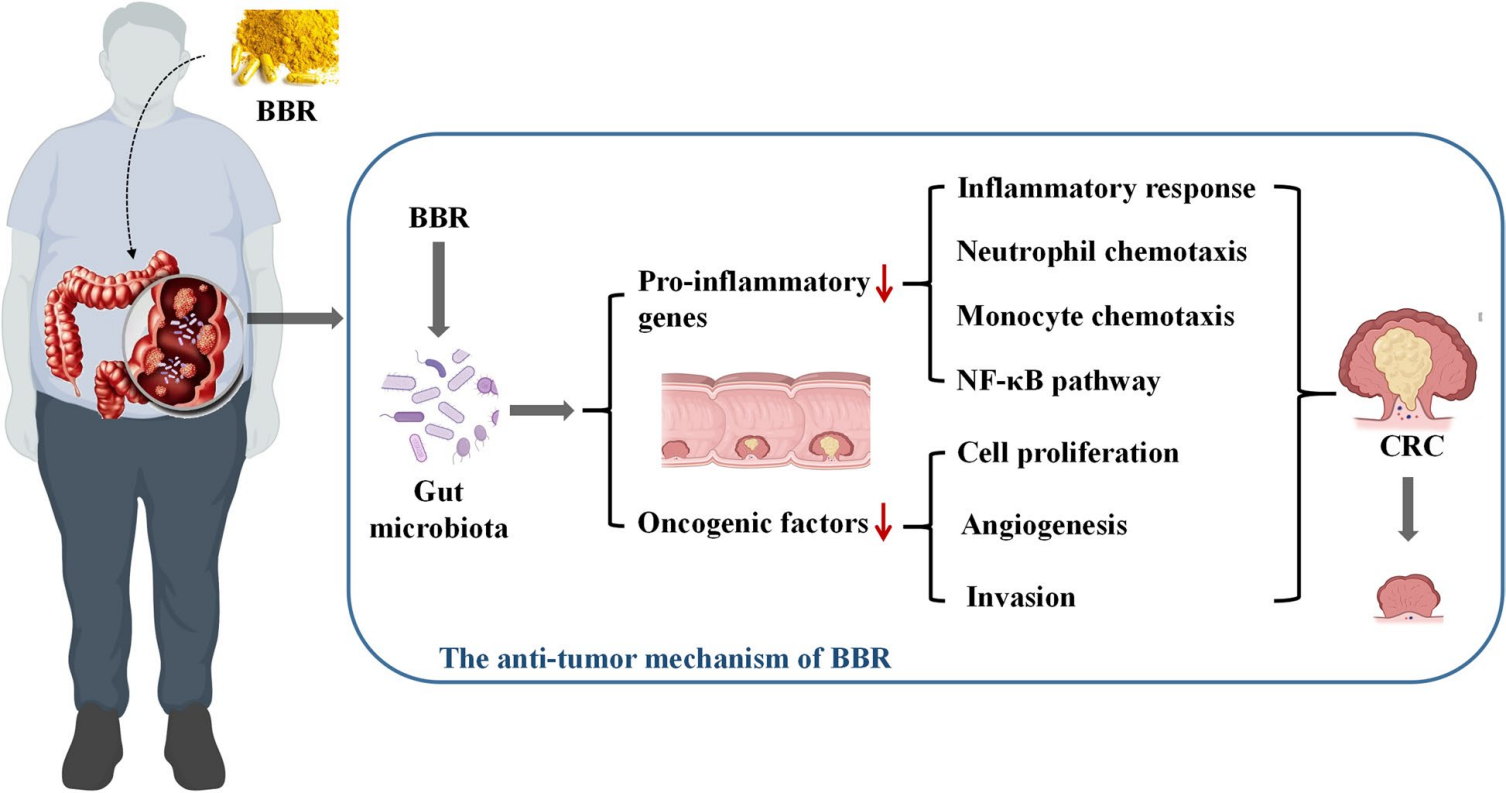
The anti-tumor mechanism of BBR. Berberine inhibits intestinal carcinogenesis by suppressing intestinal pro-inflammatory genes and oncogenic factors through modulating gut microbiota

In conclusion, this study revealed that BBR was able to restore the enteric microbiome community in AOM/DSS mice and confirmed that regulating microbiota is one of the important pathways for BBR to inhibit the pathogenesis of CRC (Fig. 10). Given the strong association between the gut microbiota and the pathogenesis of CRC, this modality of treatment creates a comprehensive strategy for combating CRC. Further studies along this direction should be guided toward elaborating how the suppression of keystone species interacts with other members of the microbiome in the inhibition of CRC. In summary, we provide direct evidence of the anti-tumorigenic roles of the BBR treated microbiota and offer a potential novel strategy for the study of traditional Chinese medicine treatment on CRC.

## Supplementary Information

**Additional file 1.**

## Data Availability

The Microbiome sequence datasets and RNA-Sequencing datasets are available in the NCBI (https://www.ncbi.nlm.nih.gov/bioproject/PRJNA769853). The BioSample accessions of microbiome sequence datasets are SRR16298021 to SRR16298036, and the BioSample accessions of RNA-Sequencing are SRR16502285 to SRR16502290.
