# Supplementary material for: Berberine inhibits intestinal carcinogenesis by suppressing intestinal pro-inflammatory genes and oncogenic factors through modulating gut microbiota
Source: BMC Cancer. 2022 May 20;22:566. doi: 10.1186/s12885-022-09635-9 (PMC9123795; doi:10.1186/s12885-022-09635-9)
Supplement: Supplementary file 1 — Additional file 1. [file 12885_2022_9635_MOESM1_ESM.doc]

1. (Figure 2B) All western blots showing the β-catenin protein levels from the control group, the AOM/DSS group, and the AOM/DSS+BBR group. β-Actin as the internal control.


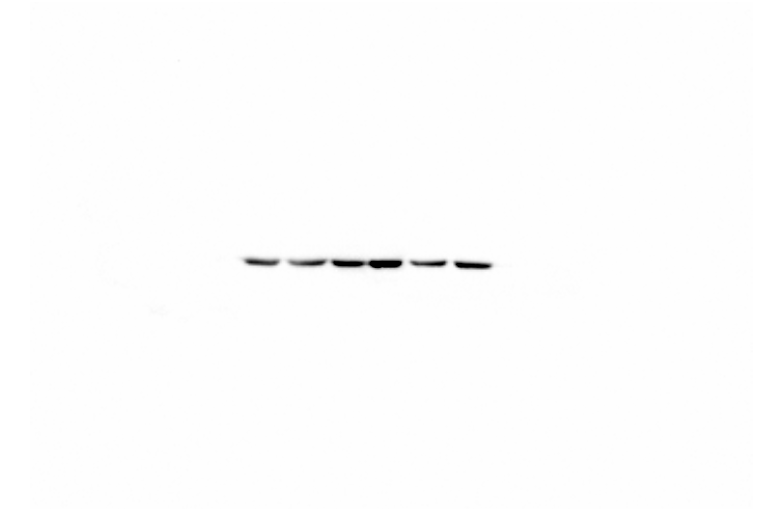

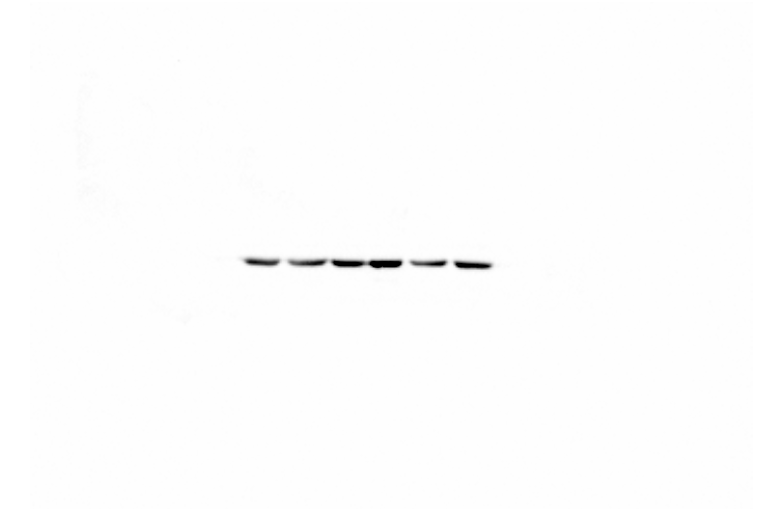


β-Actin 1-1 β-Actin 1-2


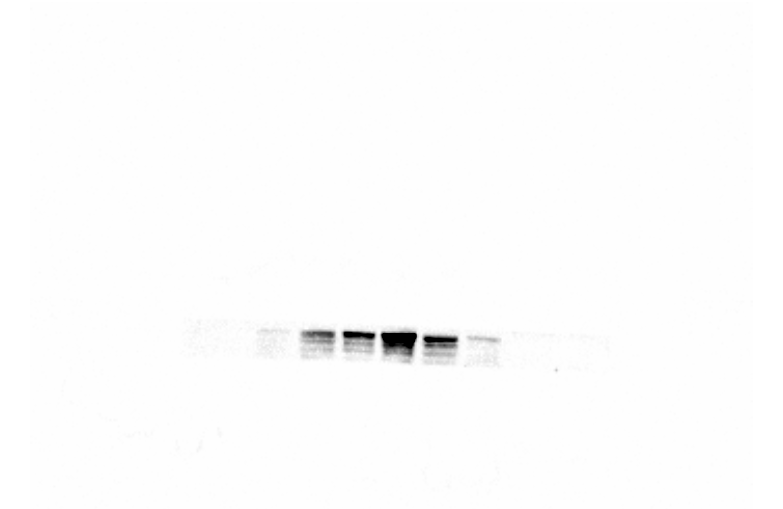

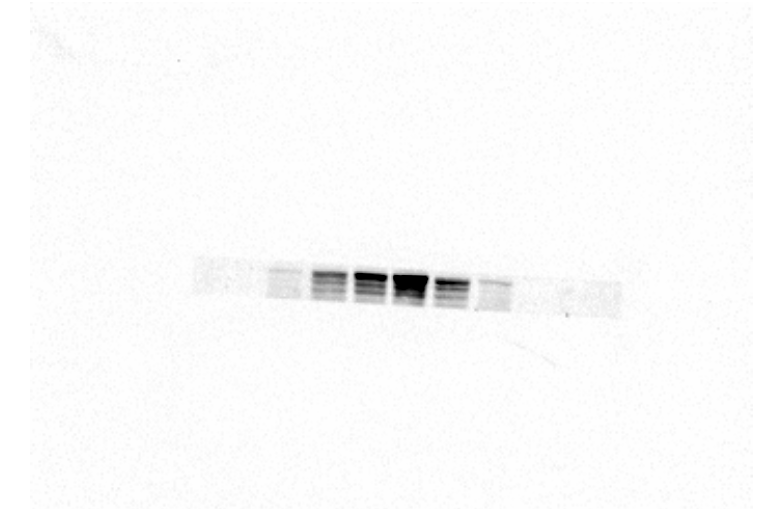

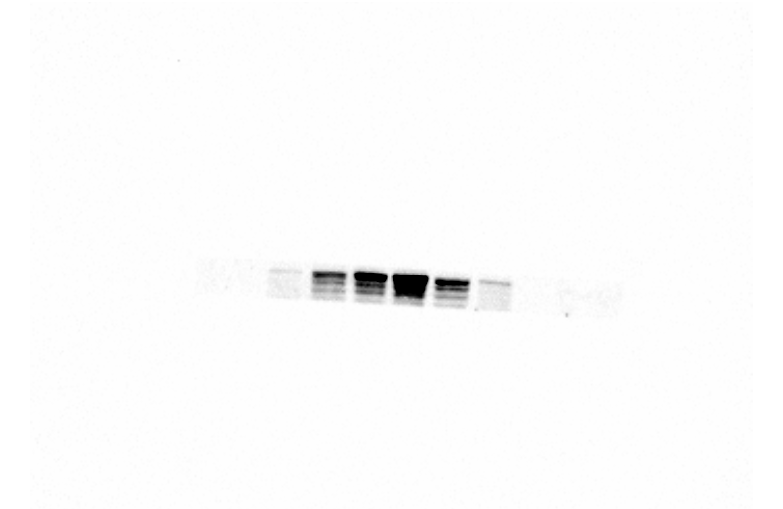


β-catenin 1-1 β-catenin 1-2 β-catenin 1-3


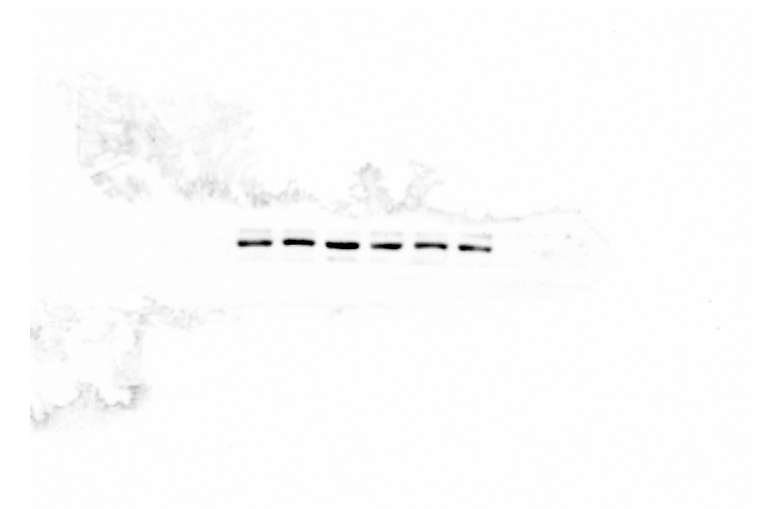

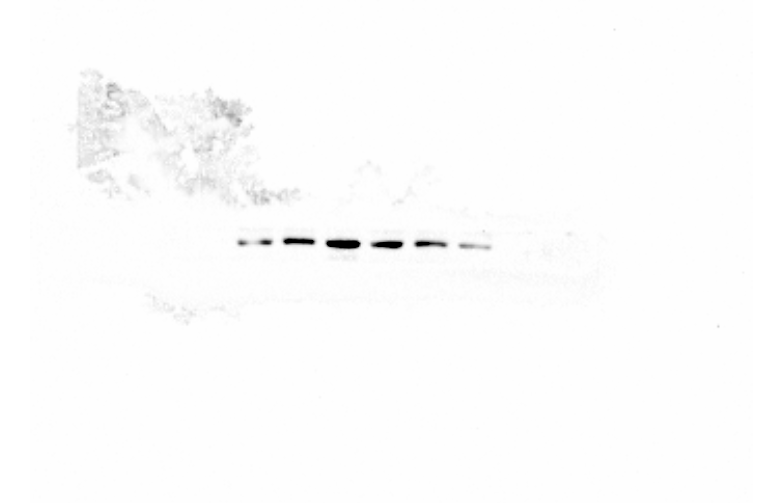


β-Actin 2-1 β-Actin 2-2


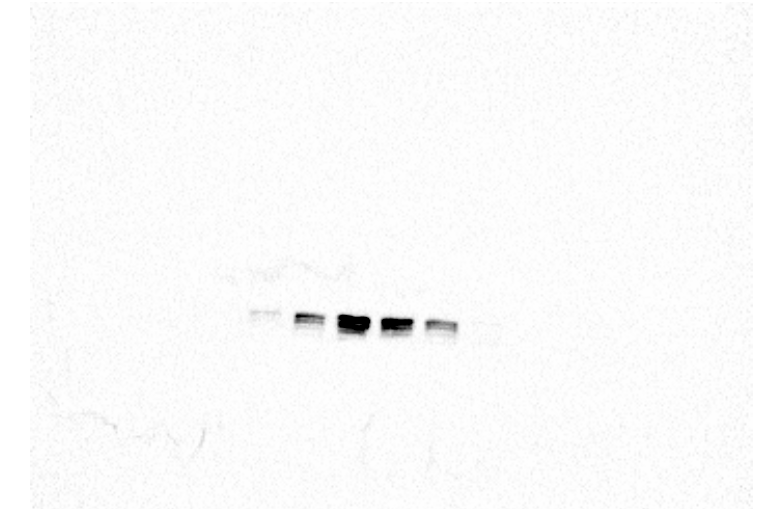

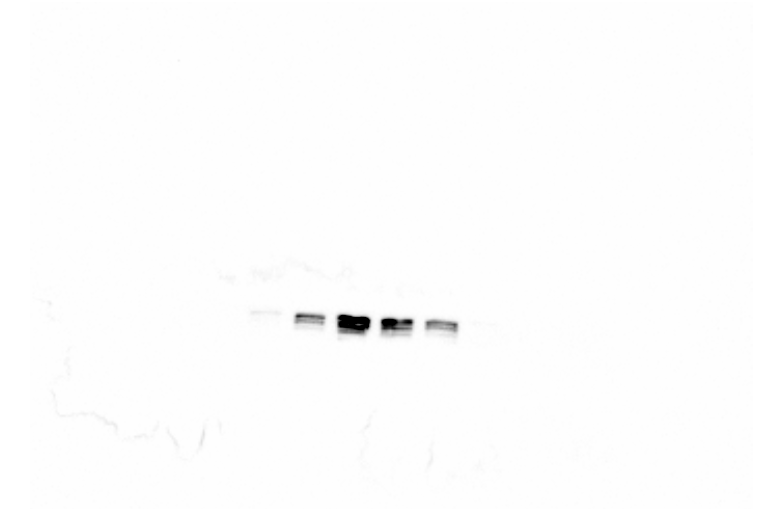


β-catenin 2-1 β-catenin 2-2


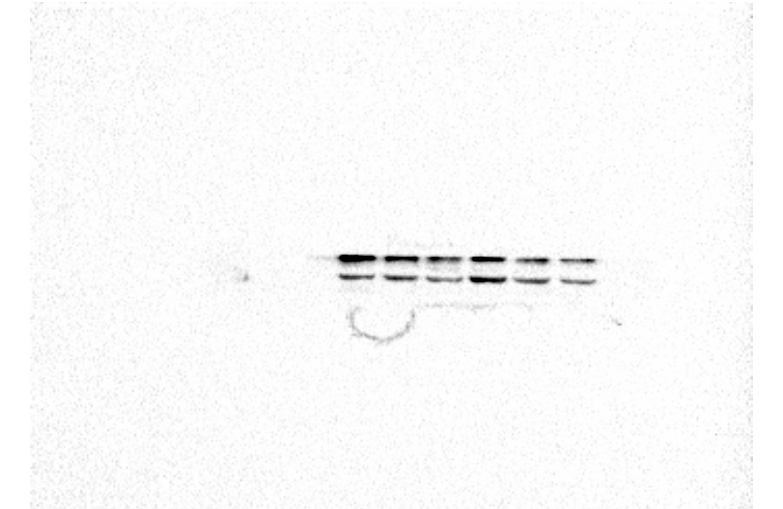

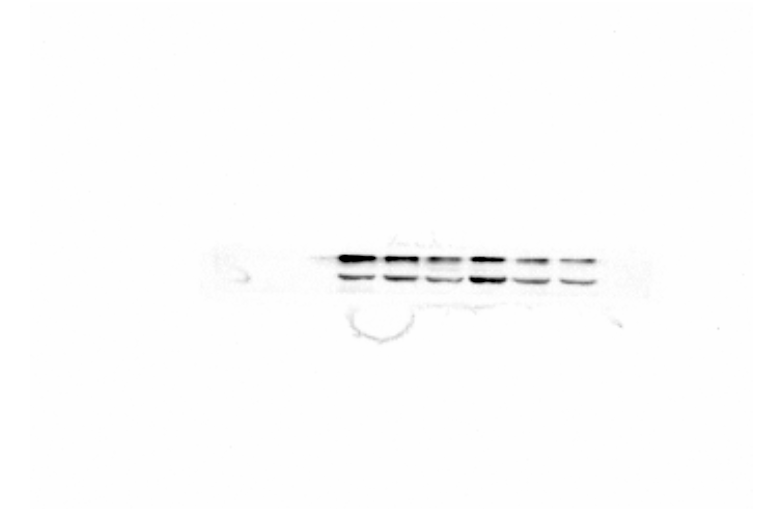


β-Actin 3-1 β-Actin 3-2


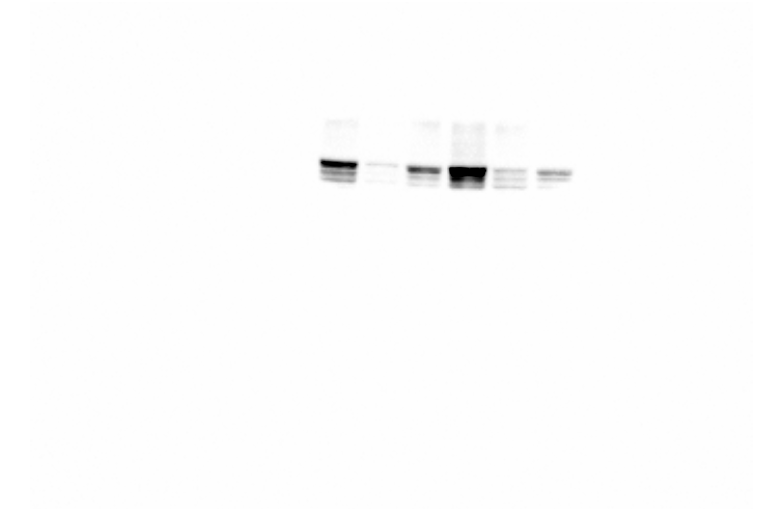

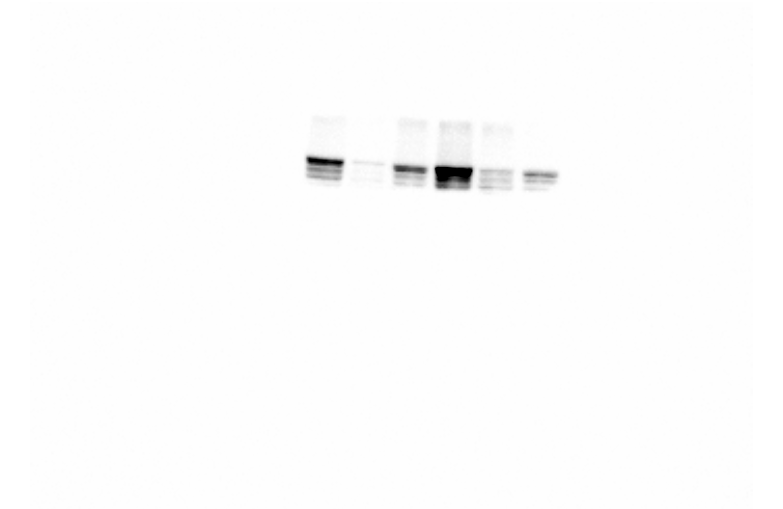


β-catenin 3-1 β-catenin 3-2

2. (Figure 7A) All western blots showing the β-catenin protein levels from the FMT (AOM/DSS) group and the FMT (AOM/DSS + BBR) group. β-Actin as the internal control.


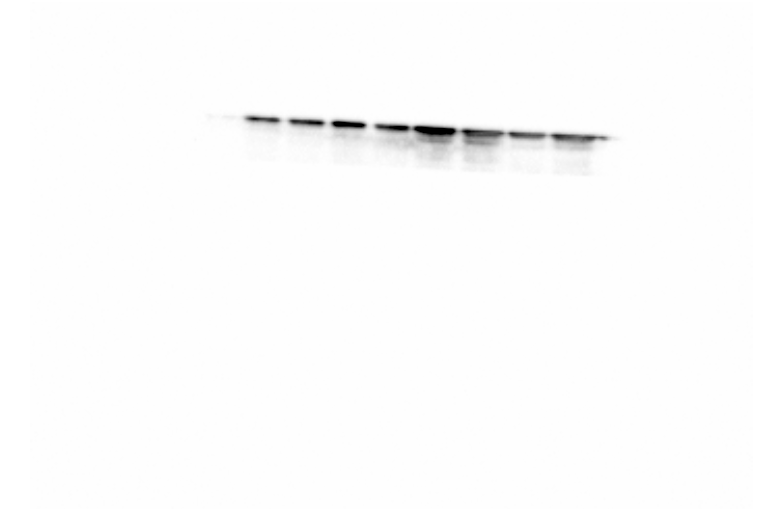


β-Actin 1


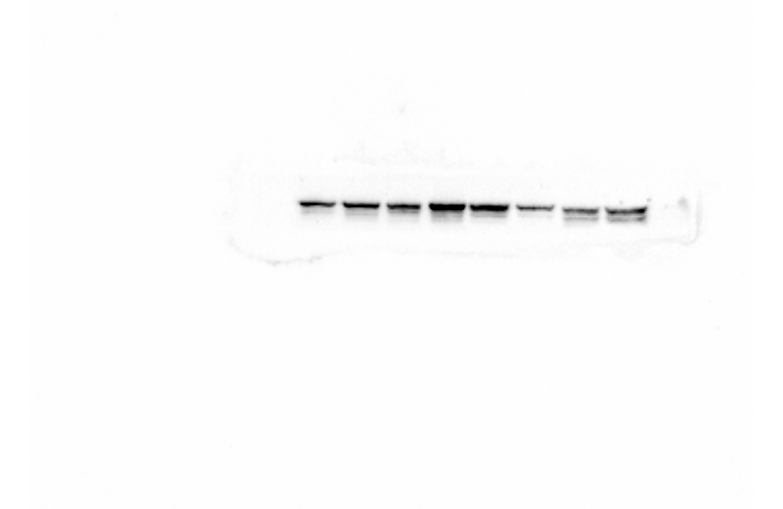

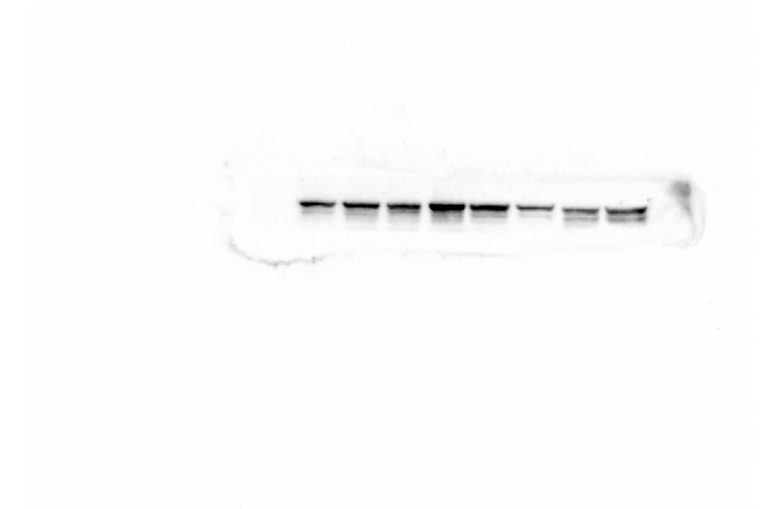

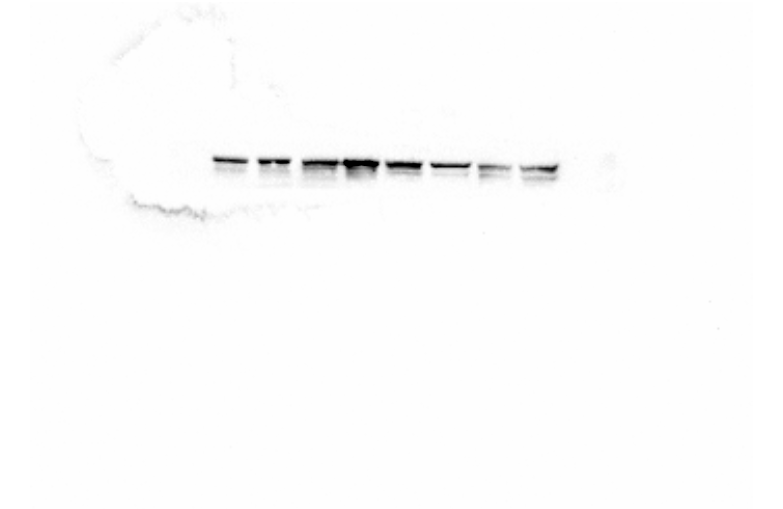


β-catenin 1-1 β-catenin 1-2 β-catenin 1-3


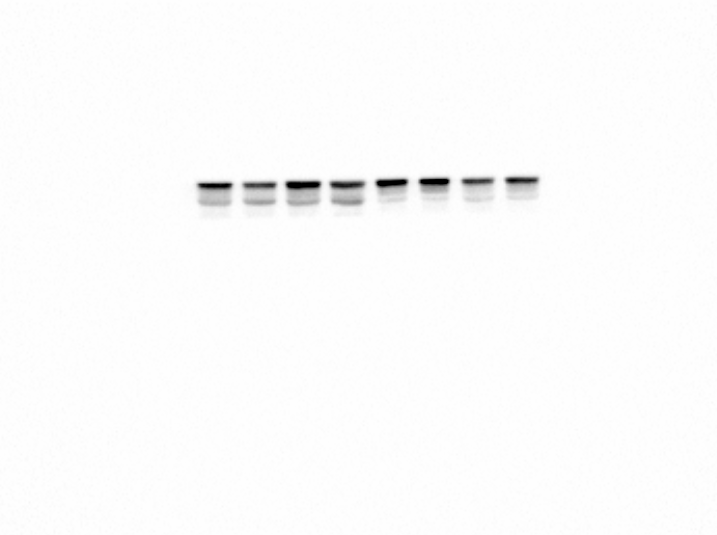


β-Actin 2


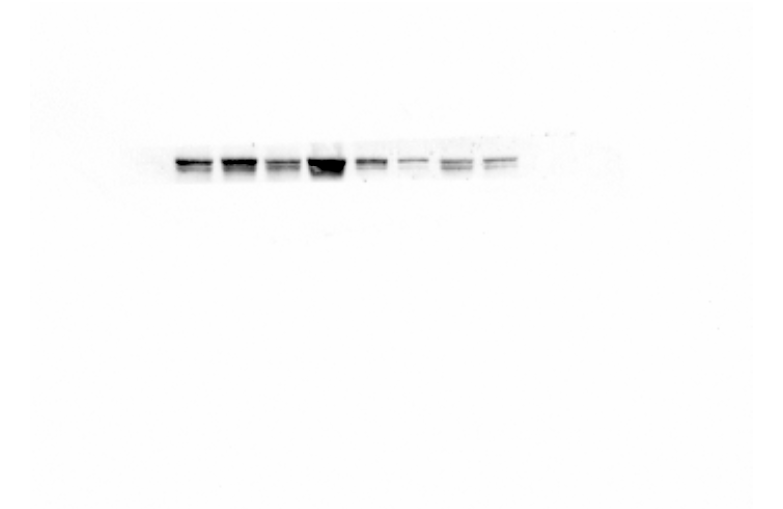


β-catenin 2


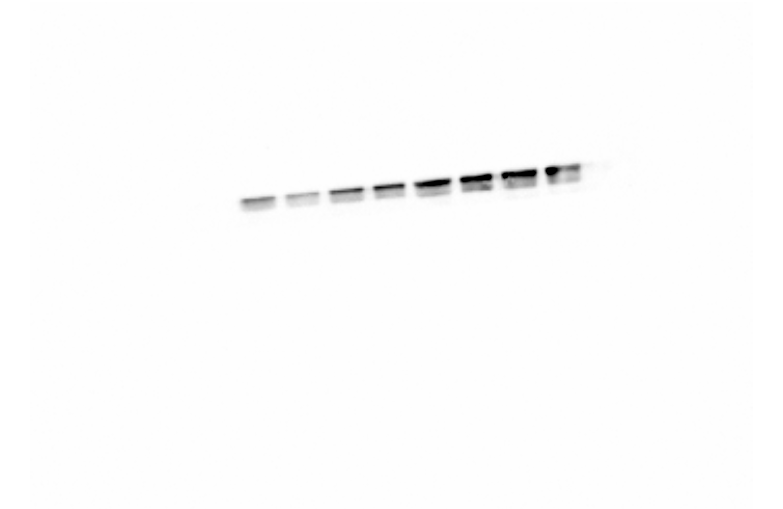

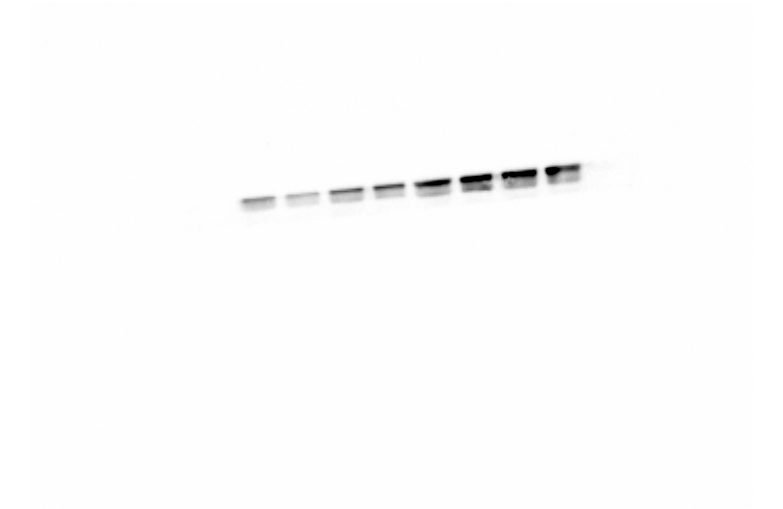


β-Actin 3-1 β-Actin 3-2


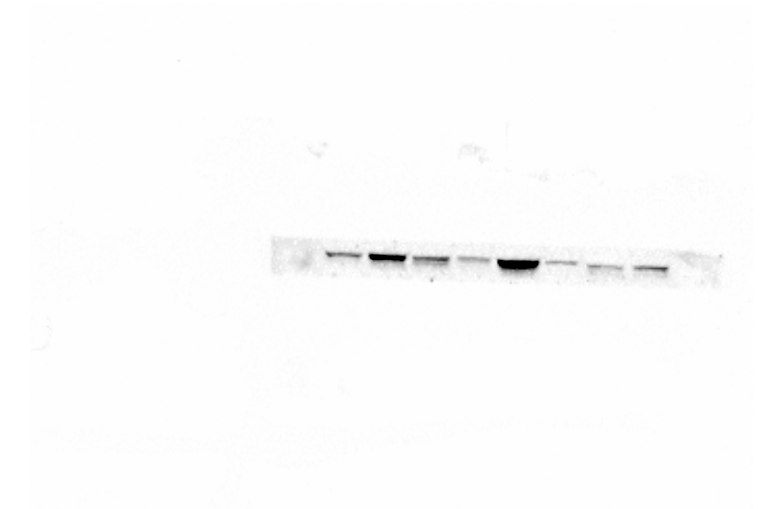

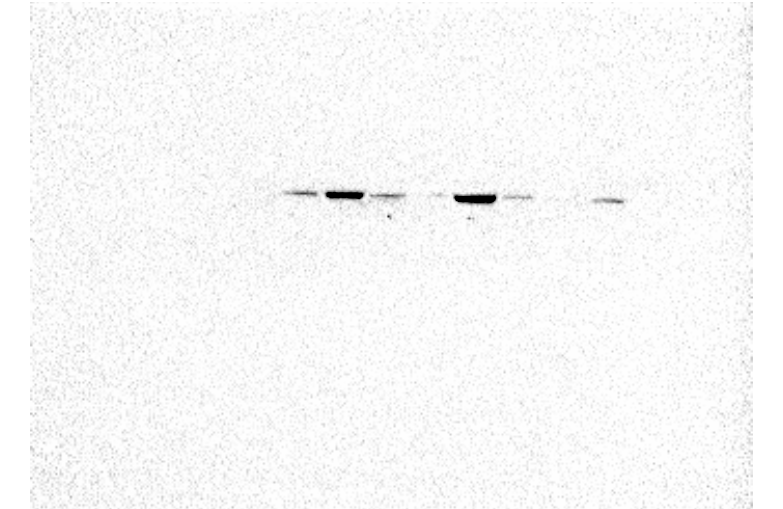


β-catenin 3-1 β-catenin 3-2

1. (Figure 7B) All western blots showing the PCNA protein levels from the FMT (AOM/DSS) group and the FMT (AOM/DSS + BBR) group. β-Actin as the internal control.


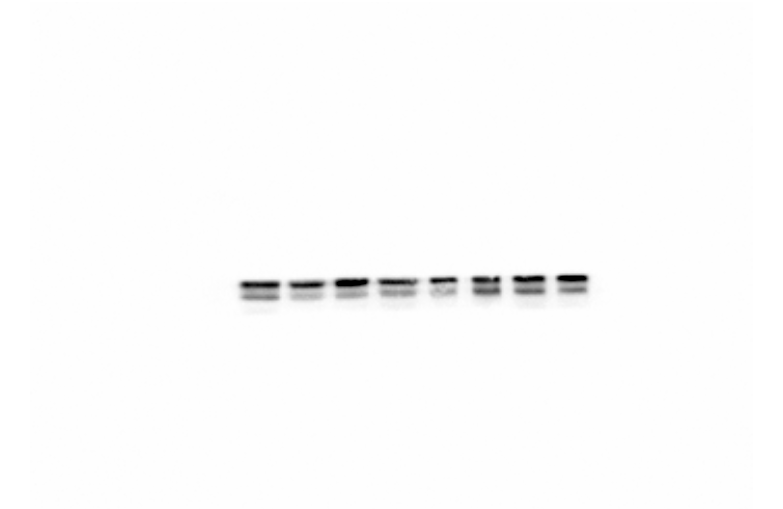

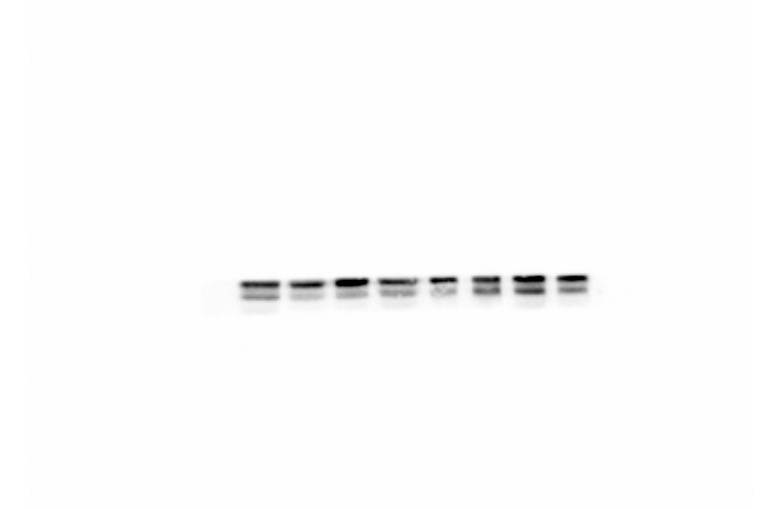


β-Actin 1-1 β-Actin 1-2


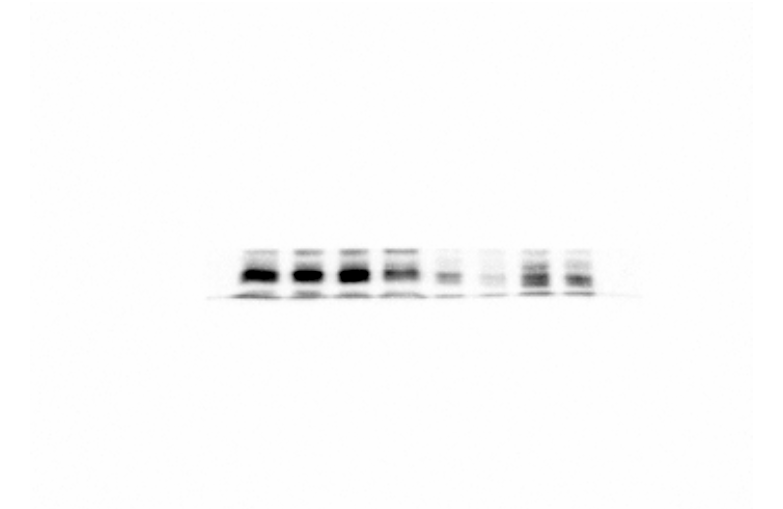

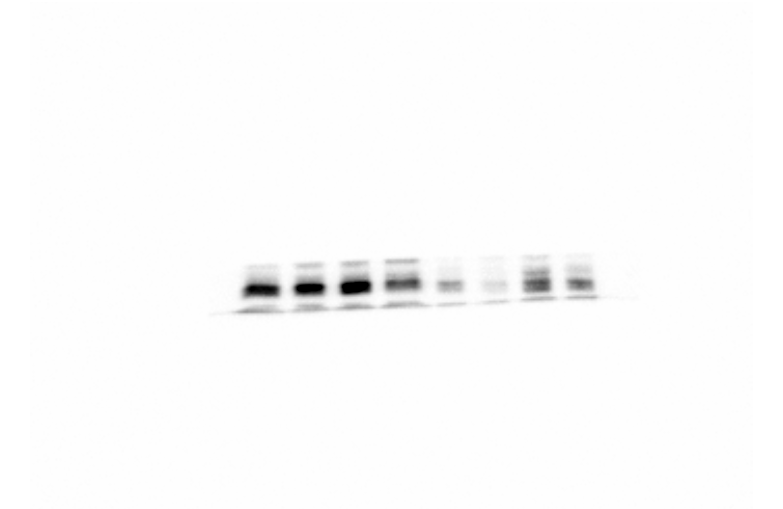

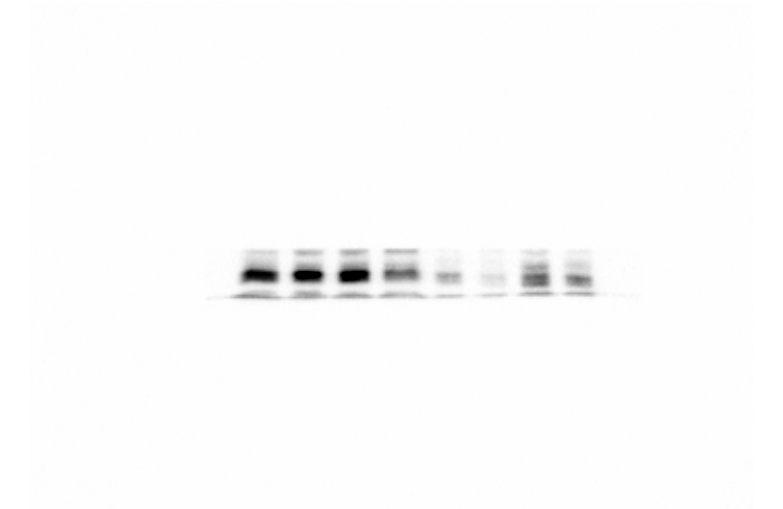


PCNA 1-1 PCNA 1-2 PCNA 1-3


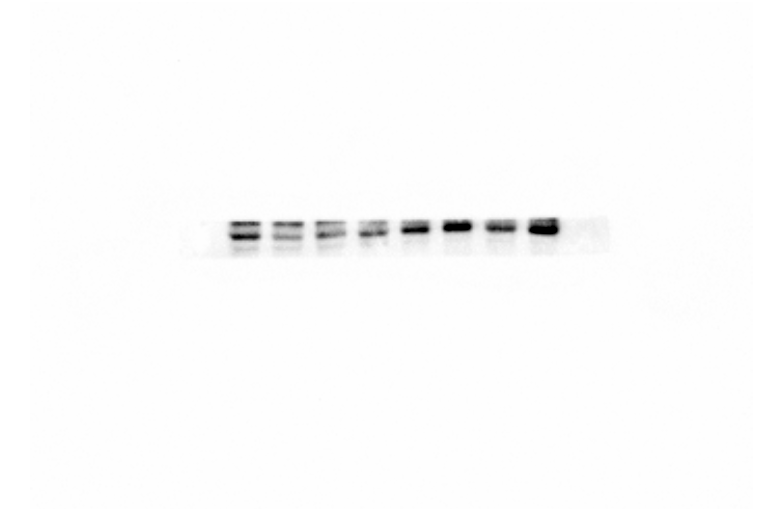

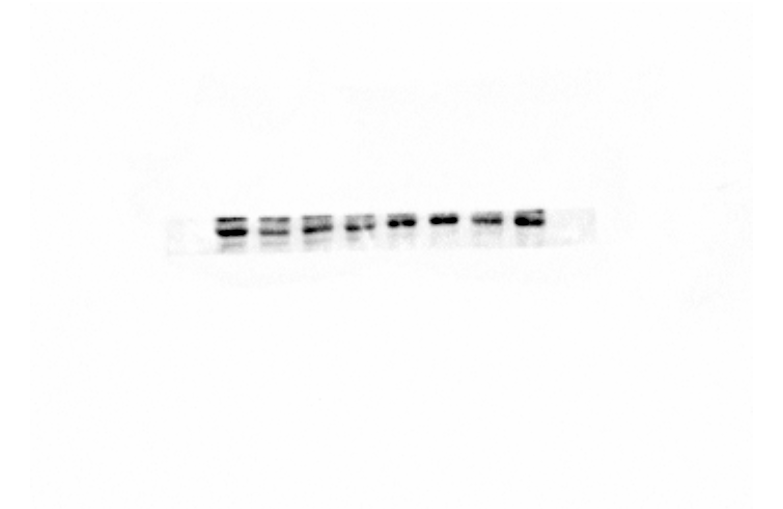


β-Actin 2-1 β-Actin 2-2


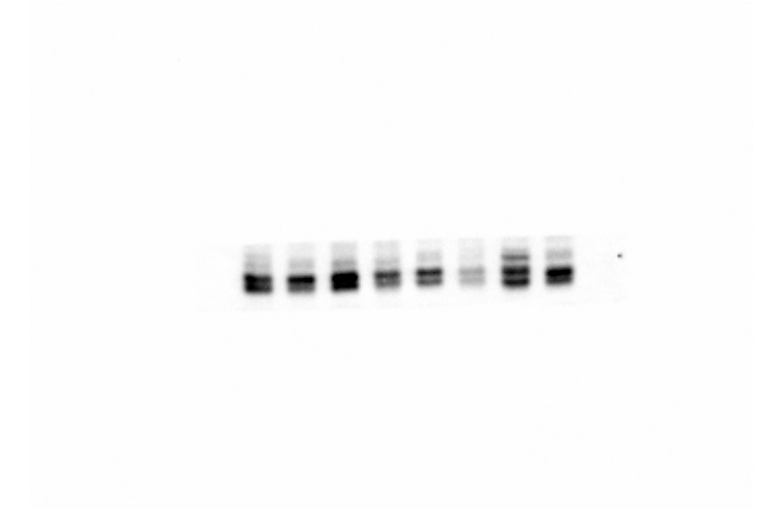

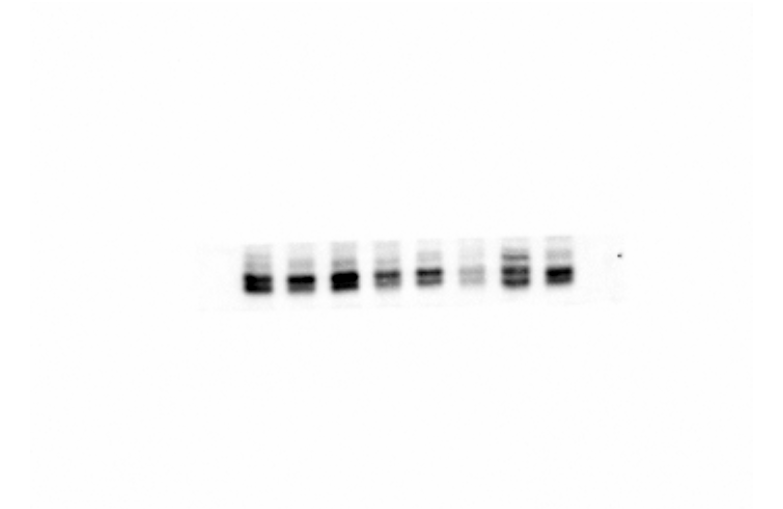


PCNA 2-1 PCNA 2-2

4. (Figure 8D) All western blots showing the IL-1b protein levels from the FMT (AOM/DSS) group and the FMT (AOM/DSS + BBR) group. β-Actin as the internal control.


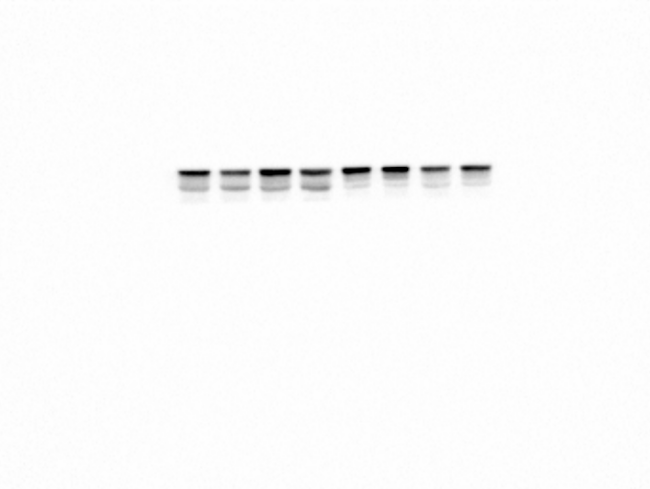


β-Actin 1


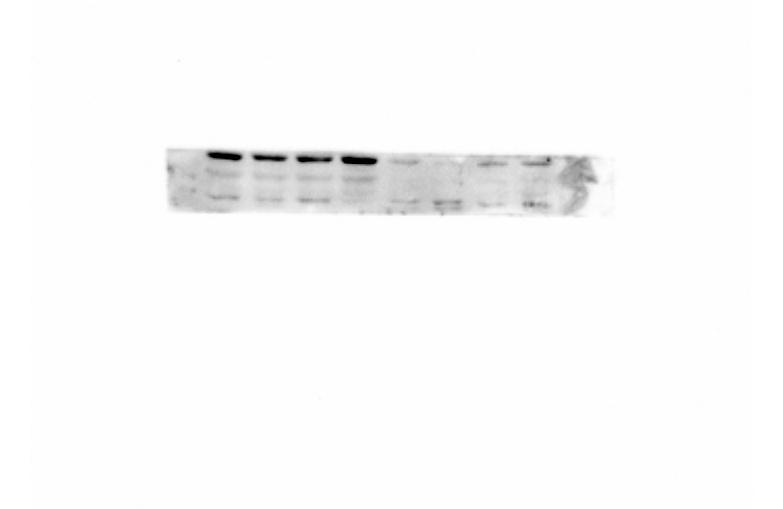

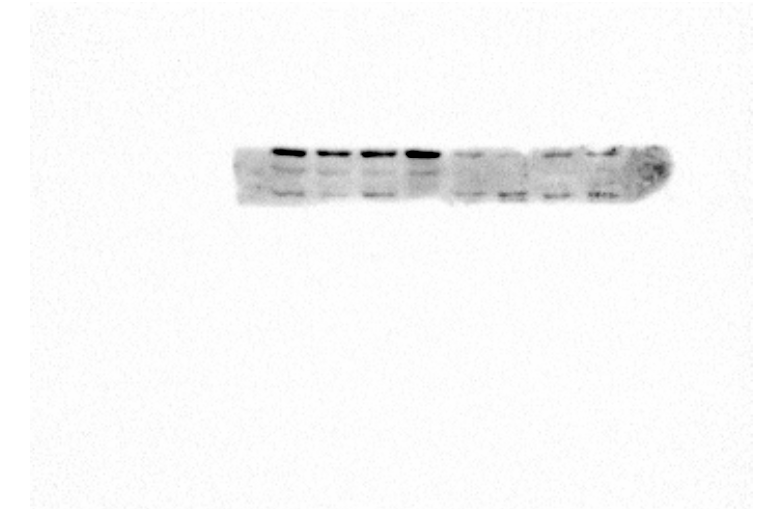


IL-1b 1-1 IL-1b 1-2


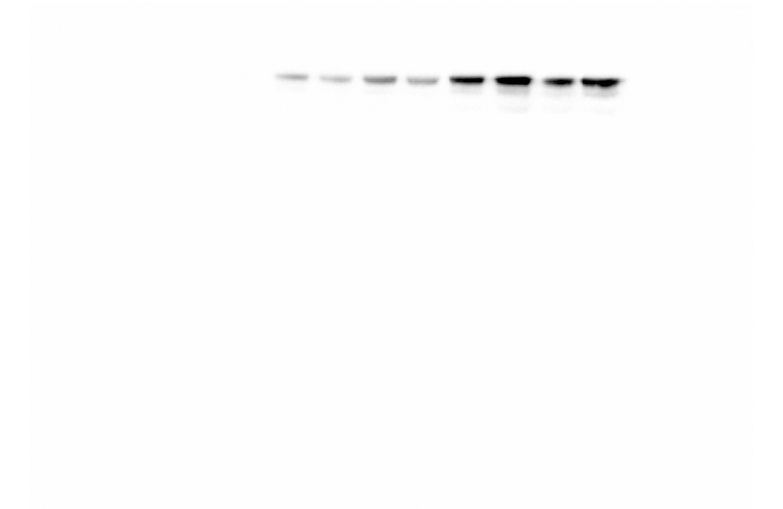

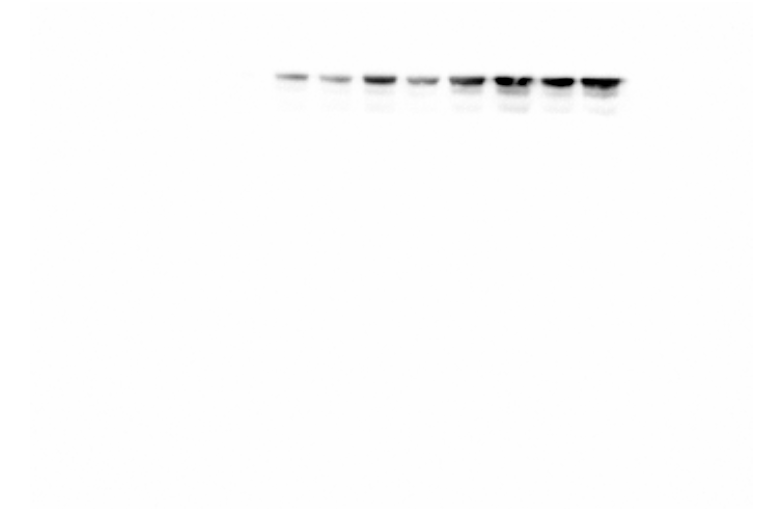


β-Actin 2-1 β-Actin 2-2


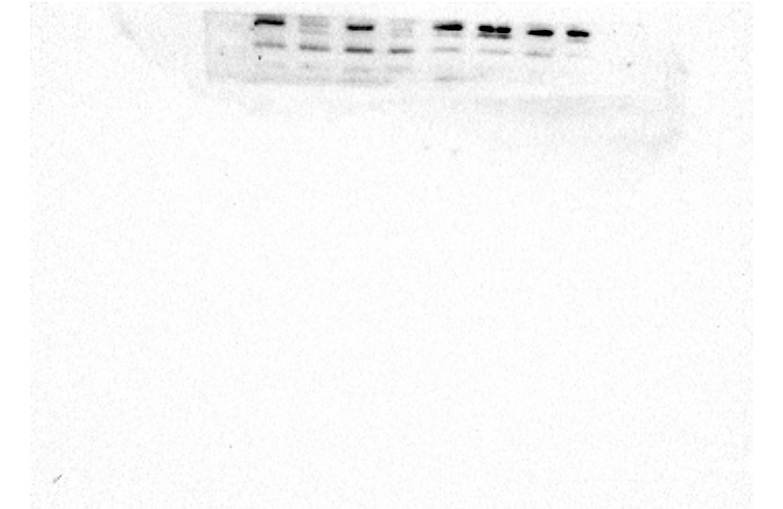

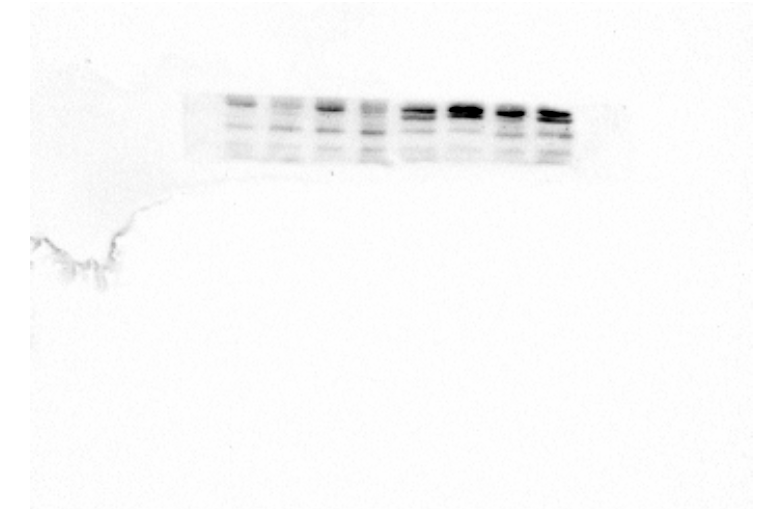


IL-1b 2-1 IL-1b 2-2

5. (Figure 8D) All western blots showing the TNF-α protein levels from the FMT (AOM/DSS) group and the FMT (AOM/DSS + BBR) group. β-Actin as the internal control.


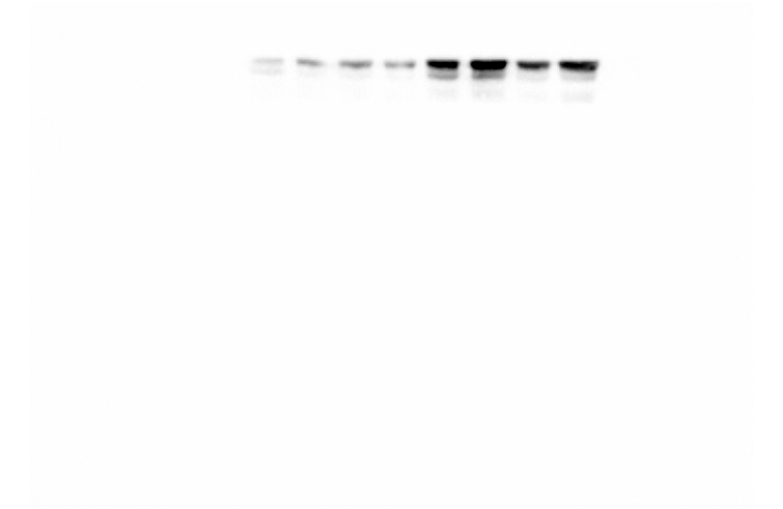

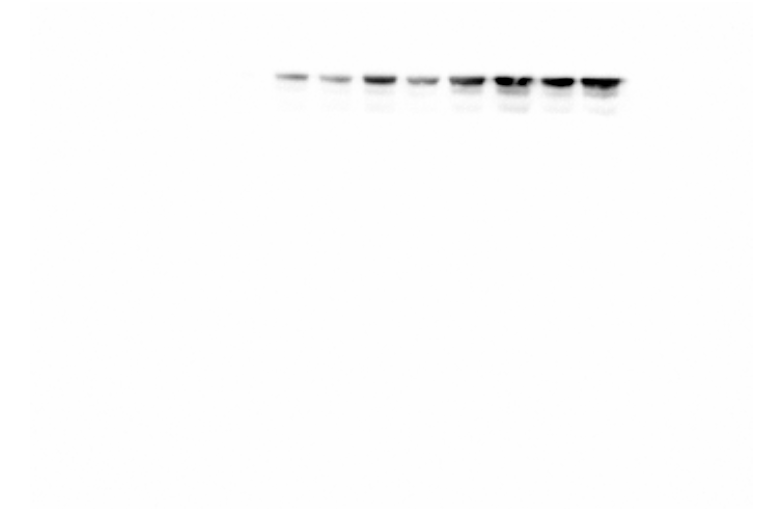

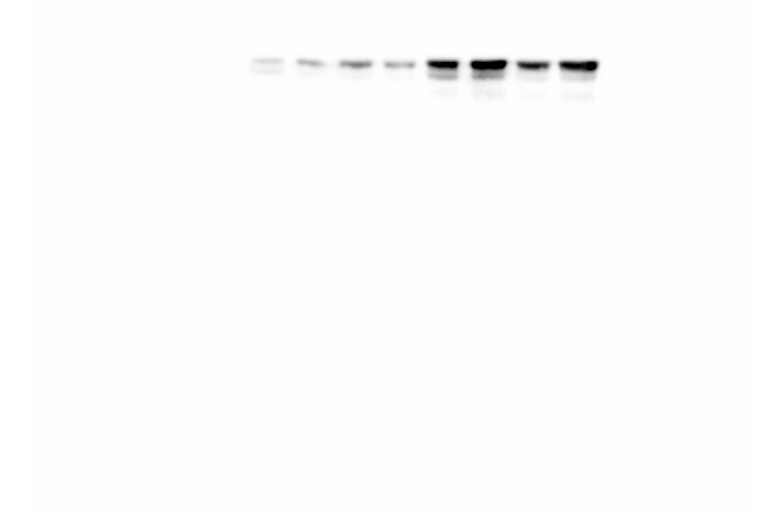


β-Actin 1-1 β-Actin 1-2 β-Actin 1-3


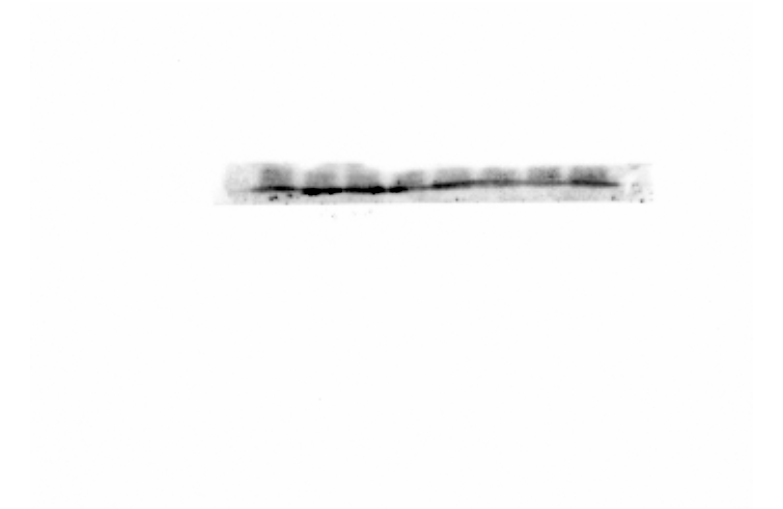

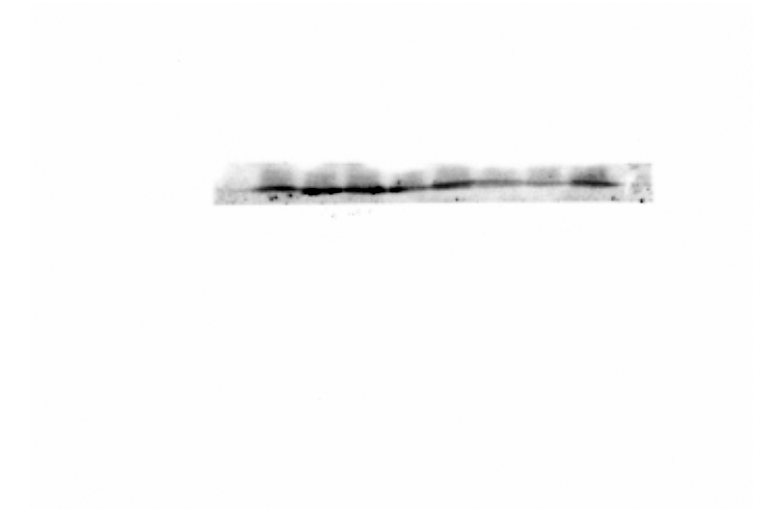


TNF-α 1-1 TNF-α 1-2


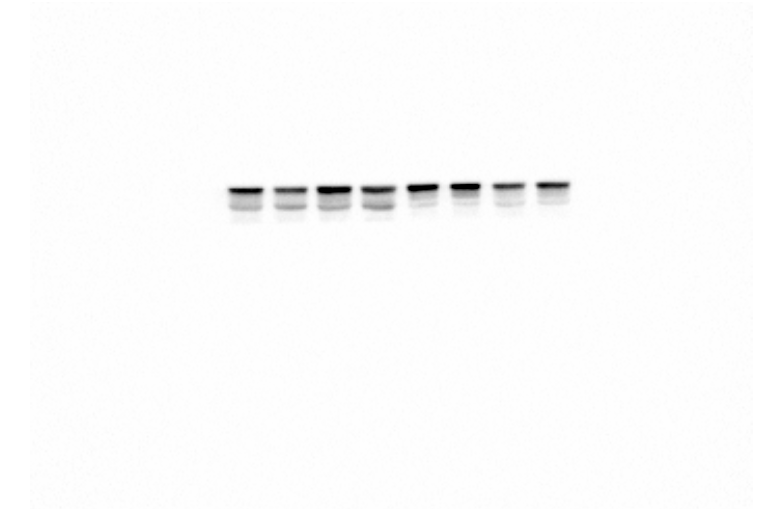


β-Actin 2


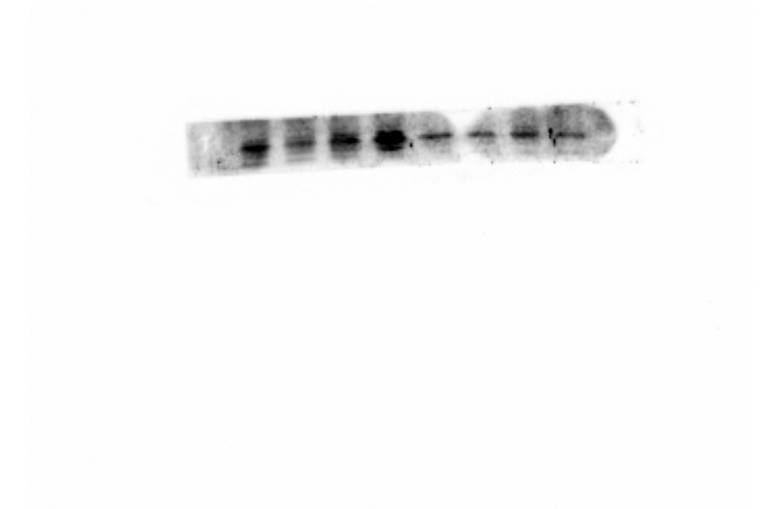


TNF-α 2


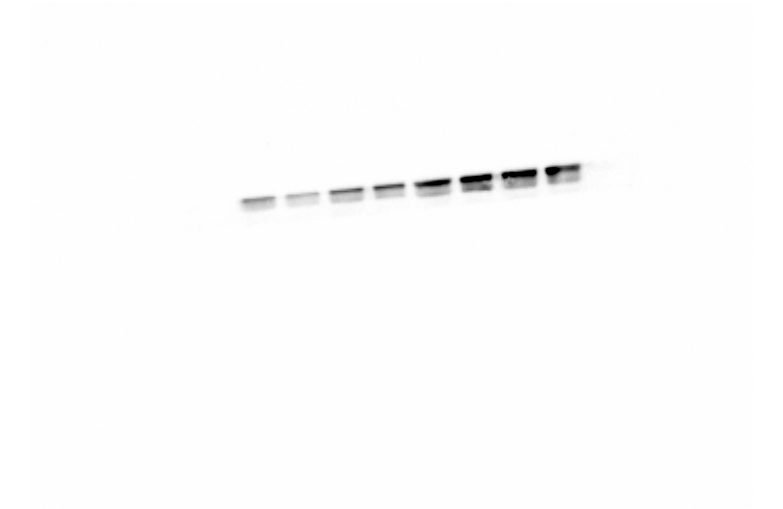

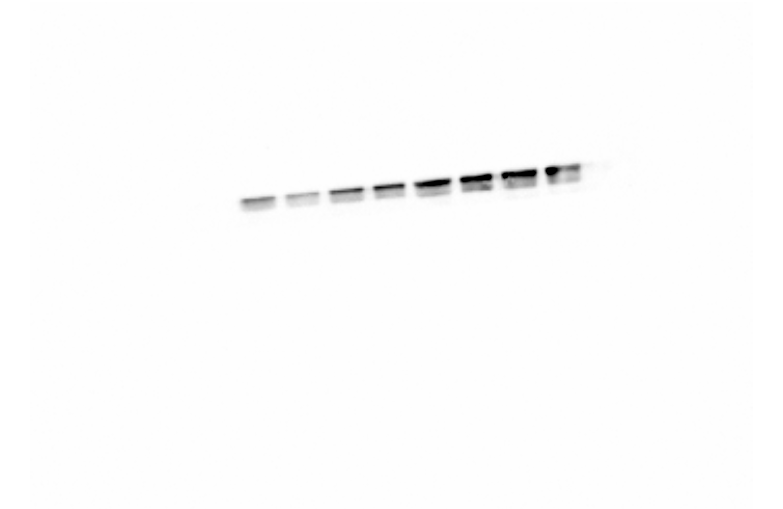


β-Actin 3-1 β-Actin 3-2


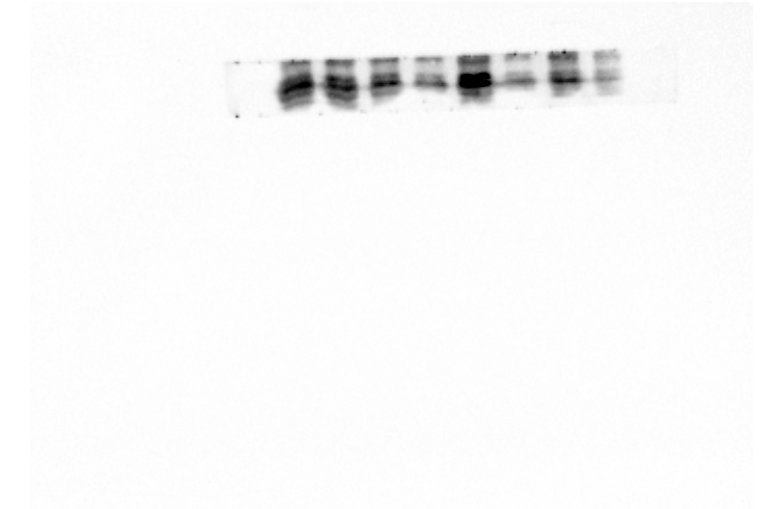

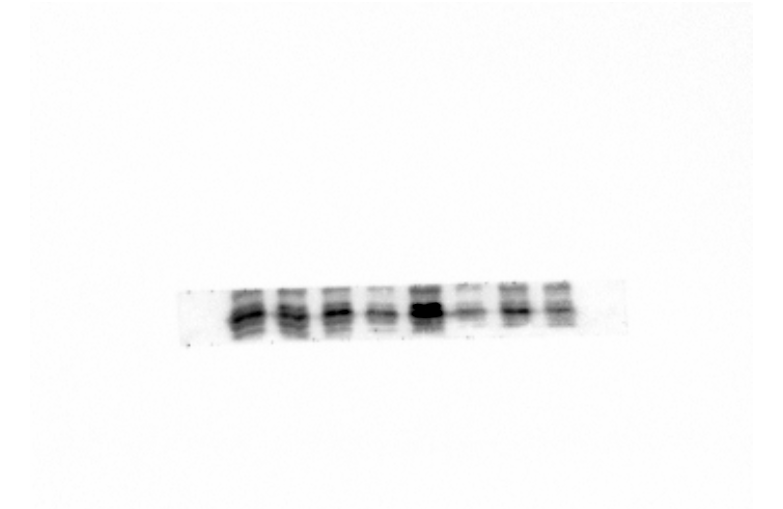


TNF-α 3-1 TNF-α 3-2

6. (Figure 8D) All western blots showing the NF-κB protein levels from the FMT (AOM/DSS) group and the FMT (AOM/DSS + BBR) group. β-Actin as the internal control.


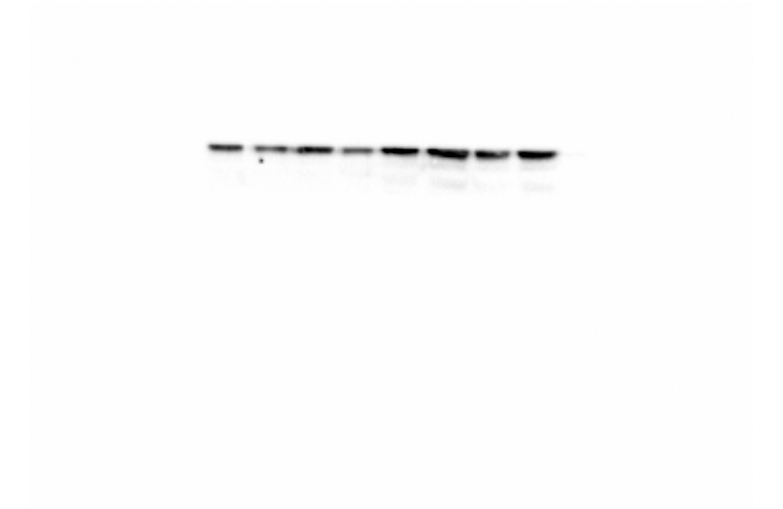

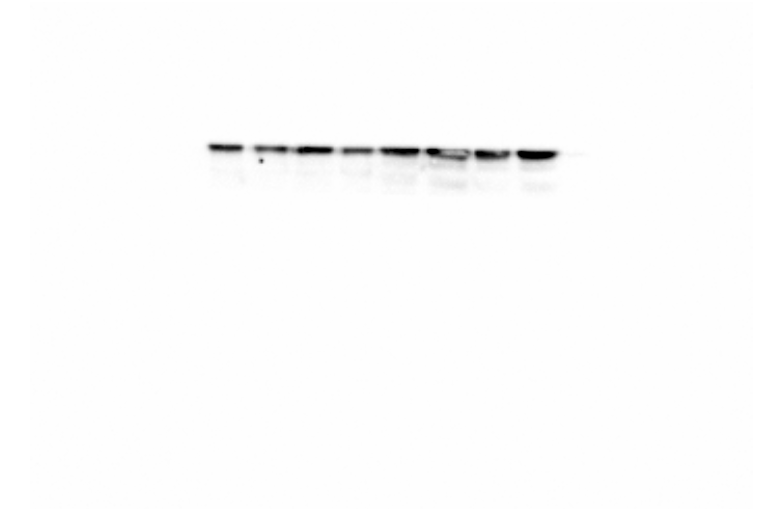


β-Actin 1-1 β-Actin 1-2


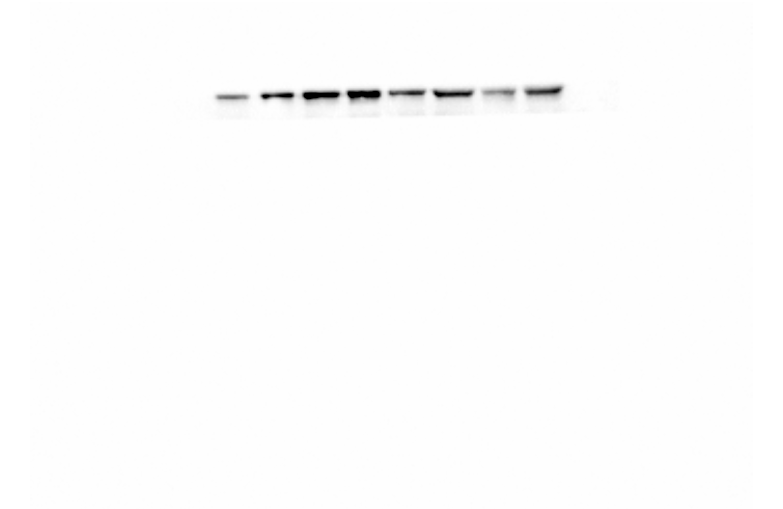

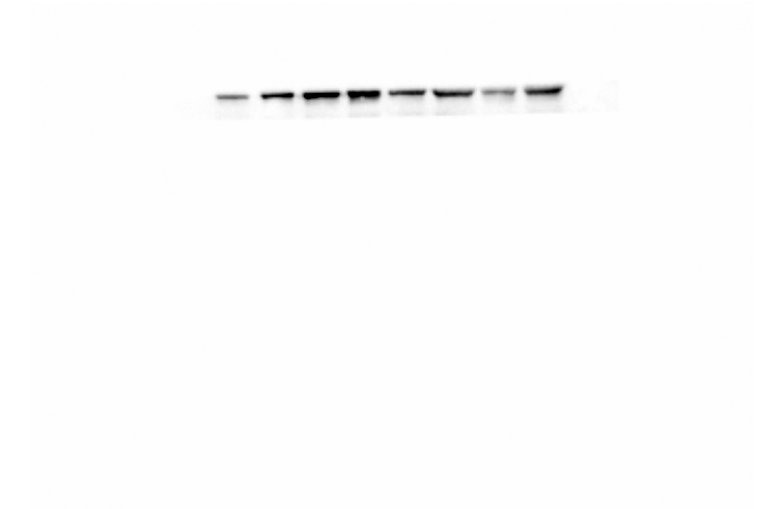


NF-κB 1-1 NF-κB 1-2


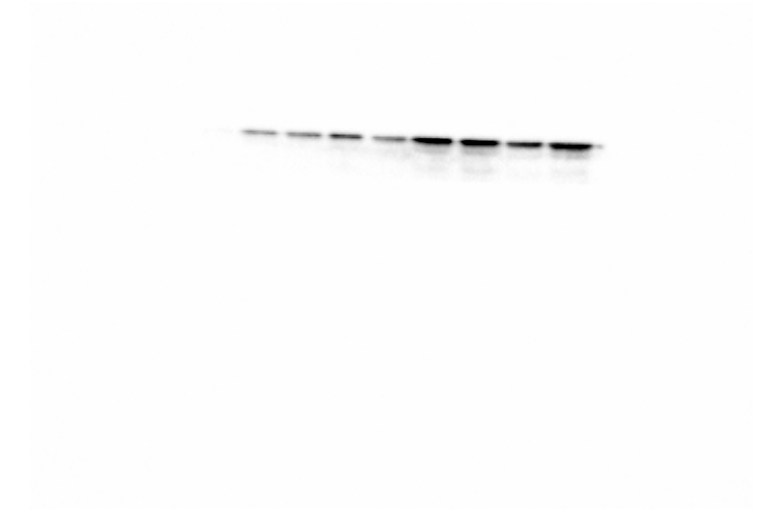

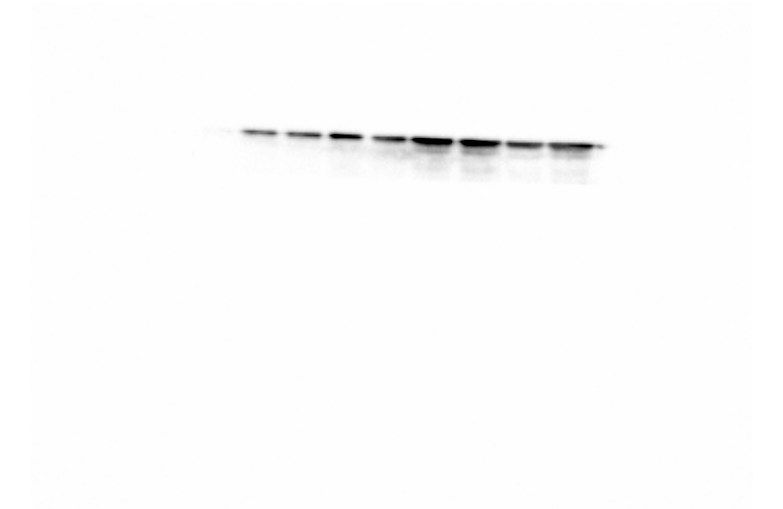


β-Actin 2-1 β-Actin 2-2


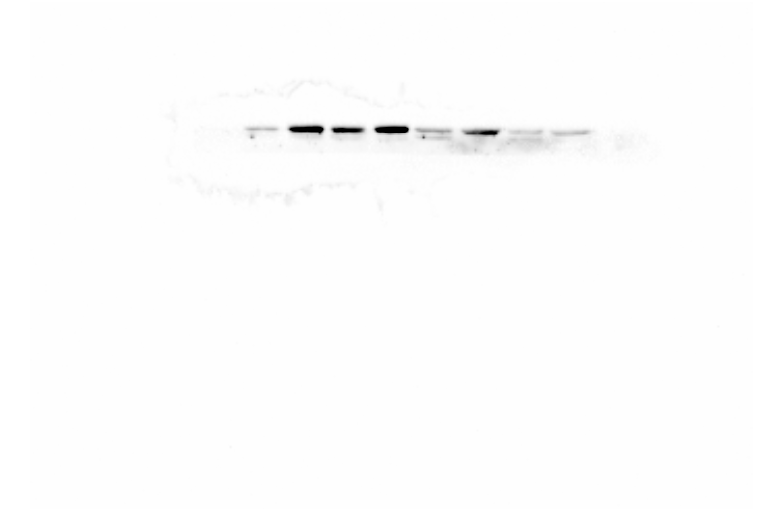


NF-κB 2


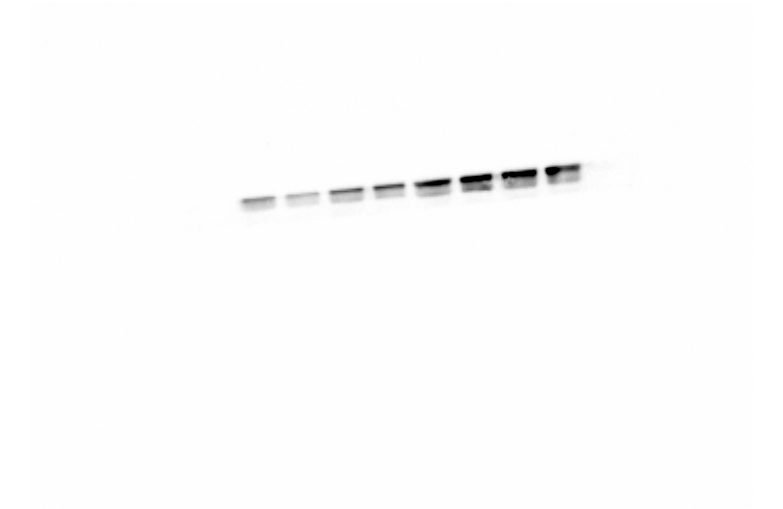

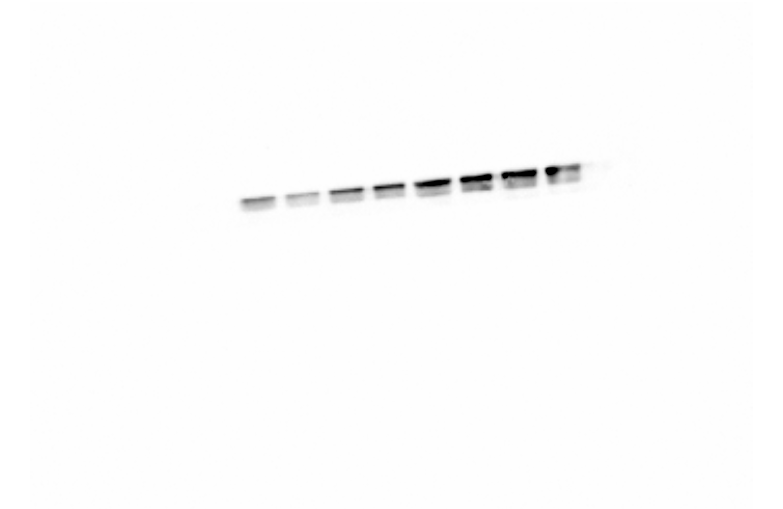


β-Actin 3-1 β-Actin 3-2


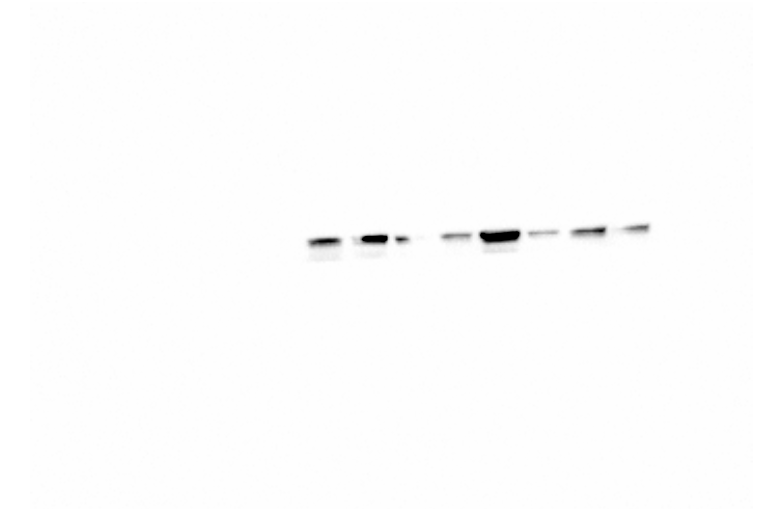

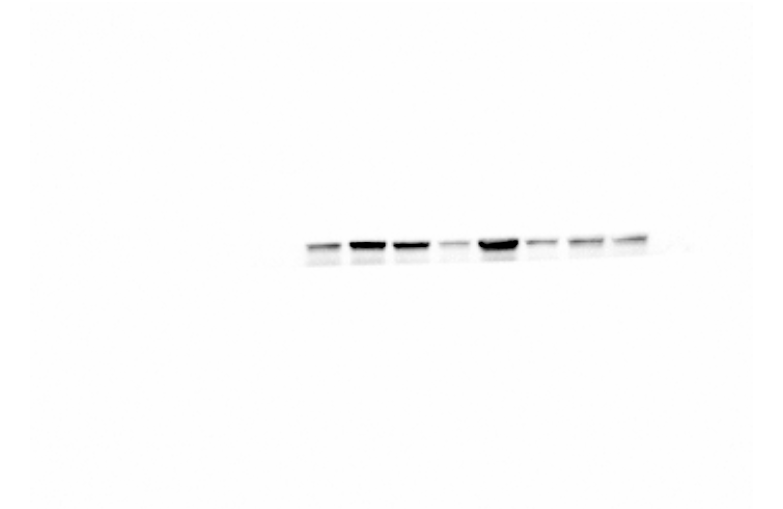


NF-κB 3-1 NF-κB 3-2
